# Integrating Bayesian variable selection with Modular Response Analysis to infer biochemical network topology

**DOI:** 10.1186/1752-0509-7-57

**Published:** 2013-07-06

**Authors:** Tapesh Santra, Walter Kolch, Boris N Kholodenko

**Affiliations:** 1 Systems Biology Ireland, Conway Institute of Biomolecular & Biomedical Research, University College Dublin, Belfield, Dublin 4, Ireland; 2 Conway Institute of Biomolecular & Biomedical Research, University College Dublin, Belfield, Dublin 4, Ireland; 3 School of Medicine and Medical Science, University College Dublin, Belfield, Dublin 4, Ireland

**Keywords:** Network inference, Bayesian statistics, Modular Response Analysis, Signaling pathways.

## Abstract

**Background:**

Recent advancements in genetics and proteomics have led to the acquisition of large quantitative data sets. However, the use of these data to reverse engineer biochemical networks has remained a challenging problem. Many methods have been proposed to infer biochemical network topologies from different types of biological data. Here, we focus on unraveling network topologies from steady state responses of biochemical networks to successive experimental perturbations.

**Results:**

We propose a computational algorithm which combines a deterministic network inference method termed Modular Response Analysis (MRA) and a statistical model selection algorithm called Bayesian Variable Selection, to infer functional interactions in cellular signaling pathways and gene regulatory networks. It can be used to identify interactions among individual molecules involved in a biochemical pathway or reveal how different functional modules of a biological network interact with each other to exchange information. In cases where not all network components are known, our method reveals functional interactions which are not direct but correspond to the interaction routes through unknown elements. Using computer simulated perturbation responses of signaling pathways and gene regulatory networks from the DREAM challenge, we demonstrate that the proposed method is robust against noise and scalable to large networks. We also show that our method can infer network topologies using incomplete perturbation datasets. Consequently, we have used this algorithm to explore the ERBB regulated G1/S transition pathway in certain breast cancer cells to understand the molecular mechanisms which cause these cells to become drug resistant. The algorithm successfully inferred many well characterized interactions of this pathway by analyzing experimentally obtained perturbation data. Additionally, it identified some molecular interactions which promote drug resistance in breast cancer cells.

**Conclusions:**

The proposed algorithm provides a robust, scalable and cost effective solution for inferring network topologies from biological data. It can potentially be applied to explore novel pathways which play important roles in life threatening disease like cancer.

## Background

We are faced with a fundamental challenge of understanding how a cell’s behavior arises from protein and gene interactions. Yet, the exact map of dynamic interactions between cellular network components is largely unknown for key cellular networks. Even for perturbations confined to single network nodes, mapping the dynamic topology of protein and gene network interactions is not straightforward. In fact, a local perturbation that is initially confined to a node rapidly propagates through the entire network, causing widespread, global changes that mask direct connections between nodes. Thus, the “reverse engineering” approaches where the connection architectures are inferred from the perturbation response data are becoming increasingly appreciated. Although reverse engineering methods such as Boolean networks [1], Bayesian networks [2,3], dynamic Bayesian networks [4,5], multivariate regression methods [6-8], linear programming [9], genetic algorithm [10] and information theoretic [11] approaches have been applied to deduce the circuitry of signaling and gene networks, all currently developed methods have significant limitations. For instance, the Boolean network based methods are found to be formidably slow, and their performance degrades with increasing network size [12]. Bayesian network methods are unable to account for feedback regulation, a hallmark of signaling networks [2]. Information theoretic approaches do not predict the directions of interactions which are important in understanding the signal flow via biological pathways [11]. A review of the advantages and limitations of most reverse engineering methods mentioned above can be found in [13].

We previously developed a method to infer network interaction maps based on steady-state responses to systematic perturbations [14,15]. This deterministic method, termed Modular Response Analysis (MRA) unravels the direction, strength and type of interactions between individual proteins and genes or between network modules that encompass several proteins or genes in a modular description. However, noise present in the data and a requirement to generate as many perturbation responses as there are nodes in the network constrain the practical applicability of this method [14]. Consequently, a stochastic equivalent of the MRA algorithm was developed to account for noise encountered in biological datasets [16,17]. However, this method is associated with high computational cost and it also is unable to analyze experimental data when the number of perturbation experiments is smaller than the number of network modules. More recently, another extension of MRA was reported, where a Maximum Likelihood approach was used to infer connection coefficients from noisy perturbation data [18].

Here, we propose a computationally efficient method which integrates the theoretical framework of MRA with a Bayesian Variable Selection Algorithm to infer functional interactions in signaling and gene networks based on noisy and incomplete perturbation response data.

## Results

### Fundamentals of the inference framework

#### Motivation

In general, network interactions can be quantified by analyzing the direct effect of a small change in one node on the activity of another node, while keeping the remaining nodes unchanged to prevent the spread of the perturbation [19]. A dimensionless quantifier (*r*_*ij*_) of this local response is the ratio of the immediate fractional change in the activity (*x*_*i*_) of node *i* to that of node *j*, (all other nodes remain fixed), and it is called the connection coefficient or local response coefficient [14], $r_{\text{ij}}=\frac{\partial x_{i}}{\partial x_{j}}$, provided that all other nodes *x*_*k*_,*k*≠*j* are kept constant.

On the other hand, the global changes (*R*_*ij*_) in node *i* occur when the other nodes become involved in the response to the perturbed node *j* through multiple interactions [14] and can be calculated using the following formula.

(1) $$R_{\text{ik}}\approx 2\left(\frac{x_{i}^{k}-x_{i}^{0}}{x_{i}^{k}+x_{i}^{0}}\right)$$

where $x_{i}^{0}$ and $x_{i}^{k}$ are the steady-state activities or concentrations of node *i* before and after perturbing parameter *p*_*k*_ respectively. Let us select node *i* and consider an n-dimensional vector $r_{i}=(r_{i1}\ldots r_{\text{in}})$ that quantifies network connections directed to node *i*. If parameter *p*_*k*_ does not directly influence node *i* the vector ***r***_*i*_ is orthogonal to *n*−1 vectors ***R***_*k*_ of the global response coefficients $(R_{1k},\ldots ,R_{\text{nk}}),k\neq i$[14,20], i.e.

(2) $$\overset{n}{\underset{j=1,j\neq i}{\sum }}r_{\text{ij}}R_{\text{jk}}=R_{\text{ik}};i\neq k;i,k=1\ldots n$$

Eq. 2 presents a precise solution to the problem of inferring the network topology (determined by connection coefficients *r*_*ij*_) from the steady-state perturbation responses [14,16,20]. It requires *n* independent perturbations to a network of *n* nodes since the matrix of global responses ***R*** must have rank *n*−1 to precisely determine connection coefficients $r_{i1},\ldots ,r_{\text{in}}$ of network edges directed to each node *i*. These relationships (Eq. 2) also assume no noise in the data. Biochemical measurements are invariably subjected to biological noise and experimental errors. Therefore, a statistical approach is more suitable for estimating the connection coefficients *r*_*ij*_ from noise corrupted global responses [16].

In a previous effort, total least square regression (TLSR) was exploited as a method for estimating the connection coefficients *r*_*ij*_ from noisy perturbation responses [16]. When the data is noisy, it is necessary to estimate the uncertainties surrounding the estimated values of *r*_*ij*_ to draw reliable inference about the nature of the corresponding interactions. Therefore, a Monte Carlo method for estimating the probability distributions of *r*_*ij*_ was proposed and successfully used to find out connection coefficients for a three-level extracellular signal-regulated kinase (ERK) cascade in a subsequent study [17]. In this case, 10^6^ sets of random realizations of the perturbation responses were drawn from normal distributions with means and standard deviations equal to those of the experimentally measured values [17]. A set of connection coefficients $r=[r_{\text{ij}},i,j=1\ldots n,i\neq j]$ was estimated from each set of perturbation responses using TLSR [17]. The values of *r*_*ij*_ calculated in this manner were used to estimate its probability distribution [17] which provides a quantitative measure of the uncertainty surrounding its estimated values. However, this method is highly computation intensive. Additionally, the proposed TLSR method [16] requires large number of perturbation experiments (typically ≥*n* for an *n* node network) which are both time consuming and expensive. Therefore, a computationally efficient method that can infer network structures using noisy data obtained from small number of perturbations (typically <*n* for an *n* node network) is required to explore cellular networks in a cost effective manner.

#### Objective

To speed up the computation process, we refrained from inferring the distributions of the connection coefficients *r*_*ij*_. Instead, we chose to infer whether node *j* directly influences node *i* or not, i.e. if there is a network connection from node *j* to *i*. In case of the deterministic MRA (Eq. 2) [14], this is a straightforward task since, by definition, *r*_*ij*_≠0 represents an edge from node *j* to node *i* and *r*_*ij*_ = 0 indicates that there is no edge from node *j* to *i*. In case of the statistical formulation of MRA [16,17], the above objective can be achieved by performing a hypothesis test such as Z-test [21] on the distribution of *r*_*ij*_ to determine whether the mean value of *r*_*ij*_ is significantly different from zero. However, this requires estimating the probability distribution of *r*_*ij*_ which is computationally expensive. To avoid the process of estimating the distributions of *r*_*ij*_, we modified the original MRA equation (Eq. 2) by introducing a new set of binary variables (*A*_*ij*_) which explicitly represent presence (*A*_*ij*_ = 1) or absence (*A*_*ij*_ = 0) of direct interaction between node *i* and *j*. Introducing these variables into Eq. 2 results in the following equation (Eq. 3), which is fully equivalent to the original MRA equation (Eq. 2),

(3) $$\overset{n}{\underset{j=1,j\neq i}{\sum }}A_{\text{ij}}r_{\text{ij}}R_{\text{jk}}=R_{\text{ik}};i=1\ldots n,k=1\ldots n,i\neq k$$

For noisy global responses (*R*_*ij*_), the above equality does not hold exactly. If we account for the difference between the left and right hand sides of Eq. 3 caused by measurement noise, then the above equation (Eq. 3) becomes,

(4) $$\;\overset{n}{\underset{j=1,j\neq i}{\sum }}A_{\text{ij}}r_{\text{ij}}R_{\text{jk}}+{∊}_{\text{ik}}=R_{\text{ik}};i=1\ldots n,k=1\ldots n_{p}^{i},i\neq k$$

Here, *∊*_*ik*_ is the difference between the left and the right hand side of Eq. 3 and $n_{p}^{i}$ is the number of performed experimental perturbations which do not directly affect node *i*. Based on the above model (Eq. 4), we propose a Bayesian Variable Selection Algorithm (BVSA) that can infer the probability of node *i* being directly influenced by node *j* (i.e. *P*(*A*_*ij*_ = 1)), without having to estimate the probability distributions of the connection coefficients (*r*_*ij*_). Additionally, in the new formulation, we relax the restrictions of required number of perturbation experiments (i.e., $n_{p}^{i}=n-1$ in case of MRA [14] and $n_{p}^{i}\geq n$ in case of stochastic MRA [16]) and allow the inference of network topology from virtually any number of perturbation experiments (i.e., $n_{p}^{i}>0$). Below, we outline the proposed Bayesian algorithm, whereas further details can be found in ‘Methods’ section and Additional file 1.

#### The proposed algorithm

**Figure 1:**
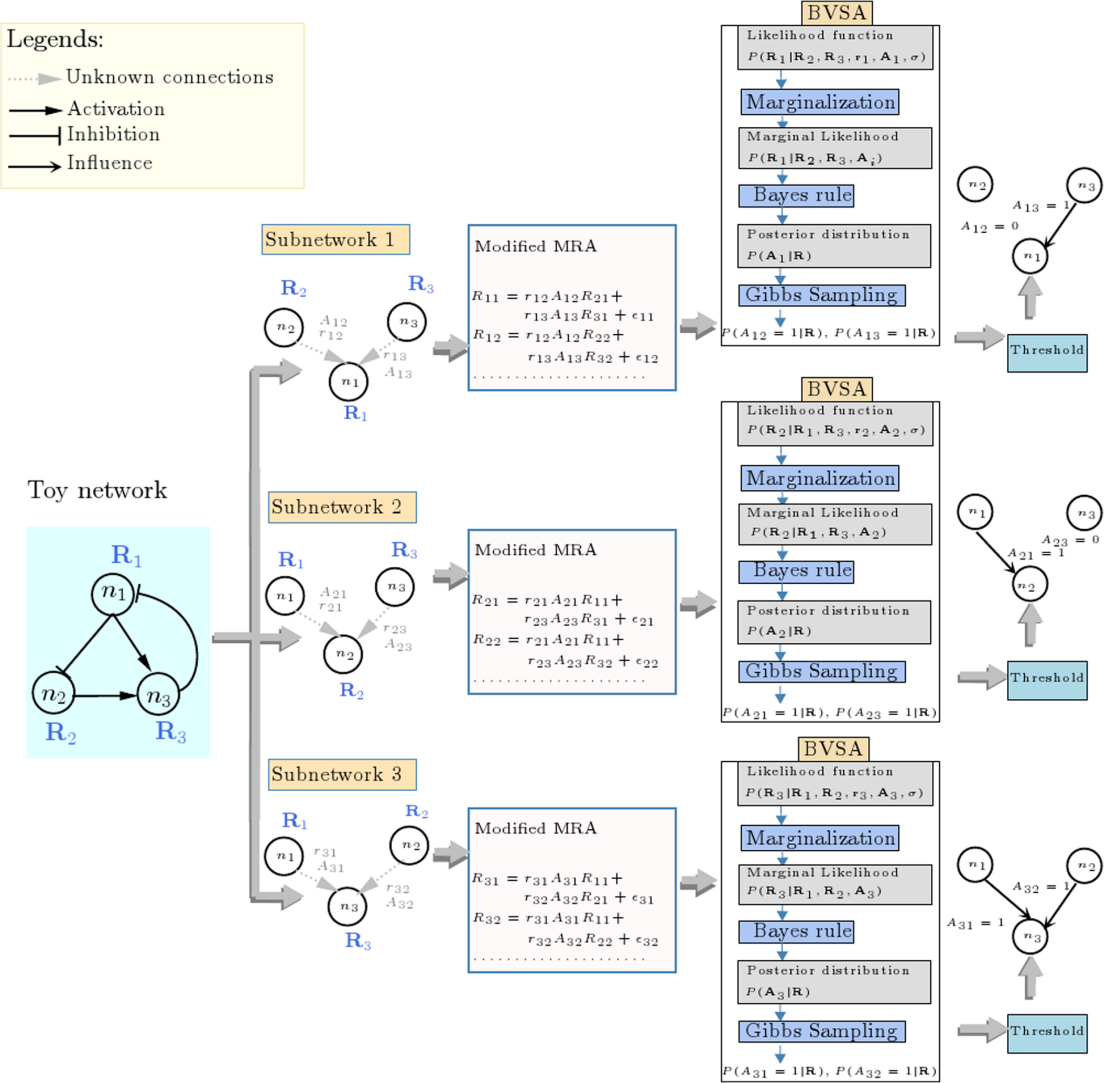
**Work flow of the Bayesian framework.** Here, we have illustrated the steps necessary for the reconstruction of a hypothetical toy network using our Bayesian framework. The toy network consists of three nodes *n*_1_, *n*_2_ and *n*_3_. The experimentally measured global responses of *n*_1_, *n*_2_ and *n*_3_ to external perturbations are denoted by $R_{1}=\{R_{11},R_{12},\ldots \;\}$, $R_{2}=\{R_{21},R_{22},\ldots \;\}$ and $R_{3}=\{R_{31},R_{32},\ldots \;\}$ respectively. For each node *n*_*i*_, we first developed a set of modified MRA equations by introducing the binary variables *A*_*ij*_ (which indicates whether *n*_*j*_,*j*≠*i* influences *n*_*i*_) into the MRA equations. Then we used the BVSA algorithm to infer the probability of *P*(*A*_*ij*_ = 1|*R*). See Additional file 1 for details of each step of the BVSA algorithm.

Eq. 4 represents a mathematical relationship between the network topology (*A*_*ij*_), the strength of each interaction (the connection coefficient *r*_*ij*_) and the measured noisy perturbation responses (*R*_*ij*_) of the network components. Here, the network topology (*A*_*ij*_), the interaction strengths (connection coefficients *r*_*ij*_) and the error (*∊*_*ik*_) caused by measurement noise are unknown variables and can be estimated from the perturbation responses (*R*_*ij*_) using statistical inference algorithms. To simplify the estimation process, we first conceptually divided a network of *n* nodes (components) into *n* numbers of smaller subnetworks, each of which consists of a node *i* and its potential regulators. The unknown variables corresponding to each of these subnetworks were then estimated independently using Bayesian statistics. In Bayesian statistics, it is assumed that our knowledge about the unknown variables is uncertain and the uncertainties surrounding these variables (*A*_*ij*_, *r*_*ij*_ and *∊*_*ik*_) are expressed in terms of their respective probability distributions. Prior to any experimental observation, these distributions are estimated based solely on our subjective assessments (assuming that little is known about the network topology a priori) and are referred to as prior distributions (see the ‘Methods’ section for a detailed description of the prior distributions of the unknown variables *A*_*ij*_, *r*_*ij*_, *∊*_*ik*_). The prior distributions were then updated based on experimentally observed data using the Bayes theorem (see Additional file 1 for details). The updated distributions are called posterior distributions. In this case, we are interested in the posterior distribution of the binary variables *A*_*ij*_ (see Methods), which represents the posterior probability of the presence (*A*_*ij*_ = 1) or absence (*A*_*ij*_ = 0) of a direct network connection from node j to node i. However, it was not possible to analytically calculate the posterior distribution of *A*_*ij*_, since it involves a normalization constant which requires calculating a very large integration (see Methods). Therefore, the posterior distributions of *A*_*ij*_ were approximated using Markov Chain Monte Carlo (MCMC) sampling (as detailed in Methods). Finally, the topology of the network was inferred by thresholding the approximate posterior distributions of *A*_*ij*_, i.e. if the posterior probability of *A*_*ij*_ = 1 is higher than a threshold value (*p*_*th*_), then we assumed that node *j* directly influences node *i*. The work flow of the proposed algorithm is graphically depicted in Figure 1 (See Methods and Additional file 1 for further details) and the source codes for a MTALAB implementation of the algorithm is provided in Additional file 2.

### Performance of the proposed algorithm for simulated and real biological networks

We studied the performance of BVSA in reconstructing both simulated and real biological networks. For simulation, we considered the Mitogen Activated Protein Kinase (MAPK) Pathway and two gene regulatory networks (GRNs) consisting of 10 and 100 genes respectively. For real biological networks we chose the ERBB signaling pathway that regulates the G1/S transition in the cell cycle of human breast cancer cells [22]. The MAPK pathway was chosen because it has many negative feedback loops which enhance robustness against perturbations [23], and its reconstruction from the steady state perturbation data poses a challenging problem. The GRNs that were chosen for this study are part of the DREAM initiative, (http://wiki.c2b2.columbia.edu/dream/index.php/Challenges, challenge 4, network 1 of size 10 and 100 categories) and are widely used for benchmarking purposes by the network inference community. The ERBB pathway was chosen due to its significance in life threatening diseases such as cancer. It has multiple feedback loops which operate via both transcriptional and non-transcriptional mechanisms and may cause resistance to anti-cancer drugs. Identifying these feedback mechanisms may provide valuable insight in developing new therapies.

The above datasets were used not only to evaluate the performance of BVSA, but also to compare its performance with many other algorithms, e.g. stochastic MRA [16,17], Sparse Bayesian Regression algorithm (SBRA) [7] and Levenberg Marquardt optimization based maximum likelihood algorithm (LMML) [18]. In case of the in-silico GRN data, we also compared the performance of BVSA with that of the winners of the DREAM challenge. We chose the above algorithms for comparison due to the following reasons. Stochastic MRA, LMML and BVSA are three different statistical formulations of the same MRA Equations [14]. Therefore, comparing these algorithms may reveal which statistical framework is more suitable for what kind of experimental data. On the other hand, SBRA and BVSA are both Bayesian Linear Regression based algorithms with different prior assumptions and network search strategies. SBRA adopted a maximum likelihood approach [7] for inferring the most likely network, whereas BVSA implements a model averaging approach which infers ‘expected’ or average networks based on the posterior probabilities of all possible networks. Hence, comparing BVSA with SBRA may also shed light on how different prior assumptions and different approaches of search strategies may affect the results.

#### Simulation study: Mitogen Activated Protein Kinase (MAPK) Pathway

**Figure 2:**
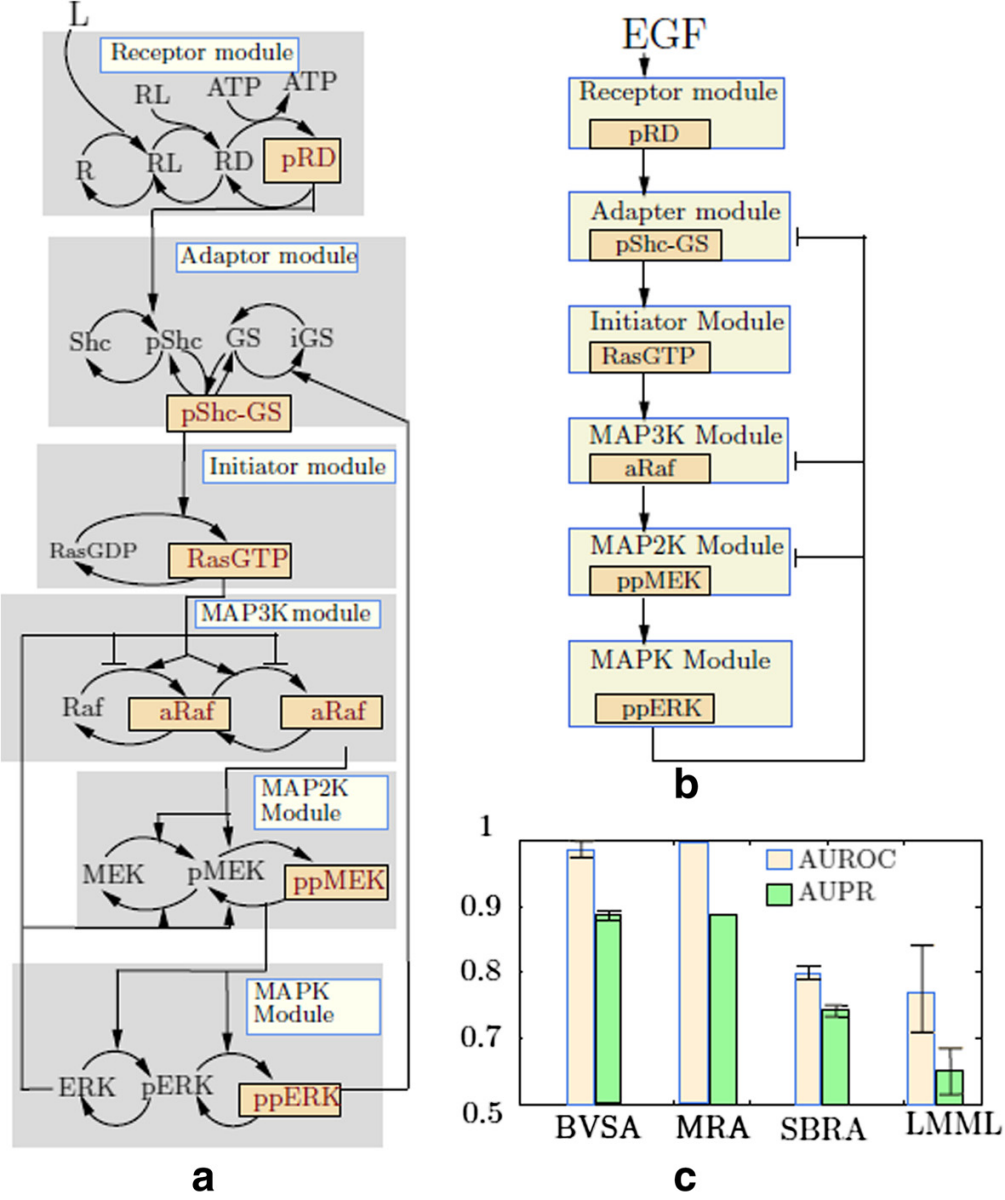
**Reconstructing the interactions among different modules of the MAPK pathway.** Panel **(a)** shows a simplified representation of the EGF pathway which activates the RAF/MEK/ERK MAPK cascade when stimulated by EGF. The conceptual modules of this pathway is highlighted in Gray. The communicating intermediates of each module are shown in yellow boxes. Panel **(b)** shows the true interactions among different modules of the MAPK pathway. In this figure, the arrowheads represent activation and the blunt arrowheads represent inhibition. Panel **(c)** shows the AUROC and AUPR values calculated from the networks which were inferred by BVSA, MRA and SBRA respectively. The error bars depict the standard deviations of the corresponding AUROCs.

MAPK pathways encompass central mechanisms of signal processing in many different eukaryotic species and participate in the regulation of a large number of important physiological processes, such as differentiation, proliferation, cell cycle and apoptosis [24]. MAPK cascades have several levels, where the activated kinase of each level phosphorylates the kinase at the next level down the cascade. The kinase of the topmost level is activated by still incompletely understood mechanisms which are usually induced by specific extracellular ligands or unspecific stress signals. For our study, we considered the Epidermal Growth Factor (EGF) induced MAPK cascade (see Figure 2(a)). EGF binds to its receptor EGFR on the outer surface of the cell membrane, resulting in its activation by means of autophosphorylation and dimerization [25]. Activated EGFR binds to and phosphorylates adapter protein Shc (among many other adapter proteins such as GAB1, IRS, Grb2 etc. which were not included in our analysis) at the cell membrane [25]. Phosphorylated Shc forms a complex with Grb2 and SOS proteins and activates the membrane bound GTPase Ras [25,26]. Activated Ras (RasGTP) triggers a MAPK cascade which consists of consecutive activation and deactivation of Raf (MAP3K), MEK (MAP2K) and ERK (MAPK) [14,25,26]. This pathway has many nested feedback loops, three of which were considered in this study. The first is a negative feedback from ERK to SOS. Activated ERK (ppERK) phosphorylates SOS causing its inactivation, which results in a decline in the activity of Ras and subsequently Raf [26]. The second is a negative feedback caused by ppERK mediated inhibition of Raf activation [26,27], and the third is a negative feedback by ppERK mediated activation of MAP2K (MEK) phosphatases [26].

The MAPK pathway was conceptually divided into six modules, where each module is a functional unit which consists of several biochemical interactions and performs one or more identifiable tasks [14]. From the top, these modules are (see Figure 2(a) and 2(b)): 

1. the receptor module which consists of the interactions leading to receptor (EGFR) activation upon ligand stimulation

2. the adapter module which consists of the phosphorylation of Shc and its complex formation with Grb2SOS

3. the initiator module which consists of the activation and deactivation of RasGDP

4. the MAP3K module which consists of the activation and deactivation of Raf

5. the MAP2K module which consists of the activation and deactivation of MEK

6. the MAPK module which consists of the activation and deactivation of ERK.

Only a single entity of each module serves as its output (referred to as “ communicating intermediates” in [14]) and carries the signal to the next module in the cascade. For the MAPK pathway, pRD (active EGFR), pShc-Grb2SOS complex, RasGTP, aaRaf (activated Raf), ppMEK and ppERK were considered to be the outputs of their corresponding modules.

We developed a mathematical model to simulate the responses of different modules of the MAPK pathway to a series of experimental perturbations.

##### Computational simulation of the MAPK pathway

Our mathematical model consists of a set of ordinary differential equations (ODE) which describe the biochemical reactions of the MAPK pathway (see Additional file 3 for details of the ODE model). Using this model we simulated different perturbations each affecting a single module. The receptor, adapter, initiator, MAP3K, MAP2K and MAPK modules were perturbed by knocking down EGFR, Shc, Ras, Raf, MEK and ERK genes respectively. Knockdown of a gene was simulated by reducing the expression level of its protein product, which depends on the efficiency of the knockdown. We assumed for illustration purpose that if a gene is knocked down with 80% efficiency then the expression level of its product protein is reduced to 20% of its original level. Each knockdown experiment was repeated three times with 40%, 60% and 80% knockdown efficiencies. After each perturbation, the MAPK pathway was allowed to reach a new steady state and the steady state responses of the output of each module was measured.

##### Network reconstruction from simulated response of the MAPK pathway

For network reconstruction, we calculated the global responses of each module to different perturbations using Eq. 1. These responses form the global response matrix ***R***. The rows of this matrix represent the network modules and the columns represent the perturbations performed on the MAPK pathway. ***R*** was then row standardized, i.e. each of its row was divided by its standard deviation. The standardization was performed to ensure equal variability in the responses of each module. The standardized global response matrix was then used to reconstruct the modular network of the MAPK pathway using BVSA. Firstly, the MAPK network was conceptually divided into six subnetworks, each of which corresponds to a certain module and its potential regulators. The topology of each subnetwork (*i*) was inferred separately, by sampling from the posterior distribution (*P*(***A***_*i*_|***R***)) of the corresponding binary variables (***A***_*i*_) using five parallel Gibbs samplers. Each of these samplers produced 200 realizations (samples) of ***A***_*i*_ in as many iterations. The convergence of these samplers are illustrated in Additional file 4: Figure S1. We rejected 20% of the initial samples drawn by each sampler as burn-ins and used the rest of the samples to estimate the probabilities *P*_*ij*_ = *P*(*A*_*ij*_ = 1).

##### Evaluating the performance of BVSA

BVSA produces a probability matrix ***P*** with the elements *P*_*ij*_ representing the posterior probability that module *j* directly influences module *i*. Using the threshold probability (*p*_*th*_), the performance of BVSA was evaluated for a range of *p*_*th*_ values, starting from *p*_*th*_ = 0, gradually incremented by 0.01, up to a maximum value of *p*_*th*_ = 1. For each value of *p*_*th*_, a network model was generated and compared with the true network model shown in Figure 2(b). The comparisons were performed by calculating the true positive (TP) rate (also known as ‘recall’), false positive (FP) rate [28] and precision [29] of the inferred networks. The TP rate is the ratio of total number of the correctly identified interactions to the total number of interactions present in the true (reference) network [28]. The FP rate is the ratio of the total number of incorrectly identified interactions and the total number of possible interactions which are absent in the true network [28]. Precision is the ratio of the total number of correctly identified interactions to the total number of interactions present in the inferred network. The curve that depicts TP rate as a function of FP rate is known as Receiver Operating Characteristics (ROC) curve [28] and the curve that depicts precision as a function of TP-rate (recall) is known as Precision-Recall (PR) curve. We calculated the areas under the ROC and PR curves for each inferred network. These two quantities, denoted by AUROC and AUPR respectively, give us a quantitative representation of the accuracy of the inferred networks. Both AUROC and AUPR can have values between 0 and 1, and the closer these values are to 1 the better is the accuracy of the inferred networks, with AUROC =1 and AUPR =1 being the ideal case.

Since BVSA uses a MCMC method to approximate the posterior distribution of the network structure its accuracy depends on the approximation error. Hence, it is necessary to evaluate the robustness of BVSA against MCMC related approximation errors. This was done by executing BVSA 10000 times on the same dataset. This resulted in 10000 different probability matrices from each of which we calculated the AUROCs and AUPRs. Then we calculated the mean and standard deviations of the AUROCs and AUPRs. The mean AUROC and AUPR represent the average performance of BVSA, and the standard deviation represents the uncertainty surrounding the performance estimate. For BVSA to be robust, the standard deviations of AUROC and AUPR must be much smaller than the corresponding means. The mean AUROC and AUPR were found to be ≈0.98 and ≈0.88 and the corresponding standard deviations were ≈0.02 and ≈0.016 respectively, suggesting near perfect and highly robust performance of BVSA on the simulated data.

We compared the performance of BVSA with that of stochastic MRA [16,17], SBRA [7] and LMML [18]. Since the simulated perturbation responses are noise free, there are no uncertainties surrounding these responses. Therefore, in case of MRA, we did not perform any Monte Carlo simulation [17] and the connection coefficients were estimated from the global response matrix ***R*** using TLSR [16]. The absolute values of the estimated connection coefficients represent the topology of the reconstructed MAPK pathway. Accordingly, the AUROC and AUPR values (Figure 2(c)) were calculated by thresholding the absolute values of the connection coefficients using a set of threshold values ranging from 0 to *∞*.

Similar to MRA and LMML, SBRA infers the interaction strengths in the form of a weight matrix ***W***[7]. An element *W*_*ij*_ of this matrix represents the strength with which node *j* influences the activity of node *i*. The sign of the weights were discarded from our analysis and AUROC and AUPR values were calculated in the same way as in the case of MRA and LMML. The uncertainty surrounding the AUROC and AUPR values were estimated in the same way as in the case of BVSA (see Figure 2(c)).

##### Network reconstruction from noisy datasets

The perturbation responses simulated by the ODE model are noise free. Real biological datasets are usually contaminated with biological noises and measurement errors. We introduced biological noise and measurement errors in the MAPK pathway simulations and used the resulting noisy datasets for network reconstruction. Biological noise is caused by many factors, such as, random thermal fluctuations, Brownian motion of the biochemical molecules, genetic variability within a cell population, etc. We developed a stochastic differential equation (SDE) model to simulate the effects of some of these factors [30] (see SI) on the dynamics of the MAPK pathway. The SDE model was simulated using Stratanovich scheme and Milstein method [31]. The effect of cell to cell variability on the perturbation responses of the MAPK pathway was ignored [30] to keep the analysis within tractable conditions.

Furthermore, we added measurement errors to the stochastically simulated responses. Measurement errors in biological datasets depend on many factors ranging from inherent biological variability to sample preparation and consistent equipment accuracy [32-34]. In almost all cases, measurement errors at least partly depend on the intensity of the signal being measured [32-34]. In many genetic and proteomic measurement systems this dependence is log-linear, i.e. linear in log scale. A simple model describing the measurement error as a function of the signal intensity is shown below [32-34].

(5) $${\sigma_{e}}^{2}=\alpha_{b}+\beta_{s}\exp\;(-Y)$$

Here, $\sigma_{e}^{2}$ is the variance of the measurement error in log scale, *α*_*b*_ is the signal independent or background noise, *β*_*s*_ is signal dependent noise and *Y* is the logarithm of the signal intensity. The background noise *α*_*b*_ and the signal dependent noise *β*_*s*_ vary among different measurement systems. However, in most high throughput proteomic experiments *α*_*b*_<0.1 and *β*_*s*_<1 [32-34]. Network inference was performed for different levels of signal dependent (*β*_*s*_) and independent (*α*_*b*_) measurement errors. We started with *α*_*b*_ = 0.01, *β*_*s*_ = 0.1 and generated 10000 datasets by repeating the stochastic simulations of the perturbation experiments and then introducing random measurement errors. A network was inferred from each of these datasets using BVSA. Similar to the noise free data, we used five parallel Gibbs samplers for each module. In this case we used 500 iterations since noisy data may slow down convergence. To see whether all parallel samplers converge to the same distribution we plotted (Additional file 5: Figure S2) the log(*P*(***A***_*i*_|***R***)) for a sample dataset. The parallel samplers generally converged rapidly to the same distribution. As before, we rejected 20% of the early samples as burn ins and the rest of the samples were used to calculate the posterior edge probabilities *P*_*ij*_. A posterior edge probability matrix ***P*** was inferred from each of the 10000 datasets using BVSA. A set of AUROC and AUPR values were calculated from each ***P***. The mean and standard deviation of the resulting 10000 AUROCs and AUPRs were calculated. *α*_*b*_ and *β*_*s*_ were then gradually increase by intervals 0.01 and 0.1 respectively up to the maximum values *α*_*b*_ = 0.1 and *β*_*s*_ = 1. For each combination of *α*_*b*_ and *β*_*s*_ we repeated the above procedure and calculated the average AUROC and AUPR values and the corresponding standard deviations (see Additional file 6: Table S1 and Additional file 7: Table S2). The average AUROC and AUPR values were then compared with those calculated from the networks inferred by stochastic MRA, SBRA and LMML.

**Figure 3:**
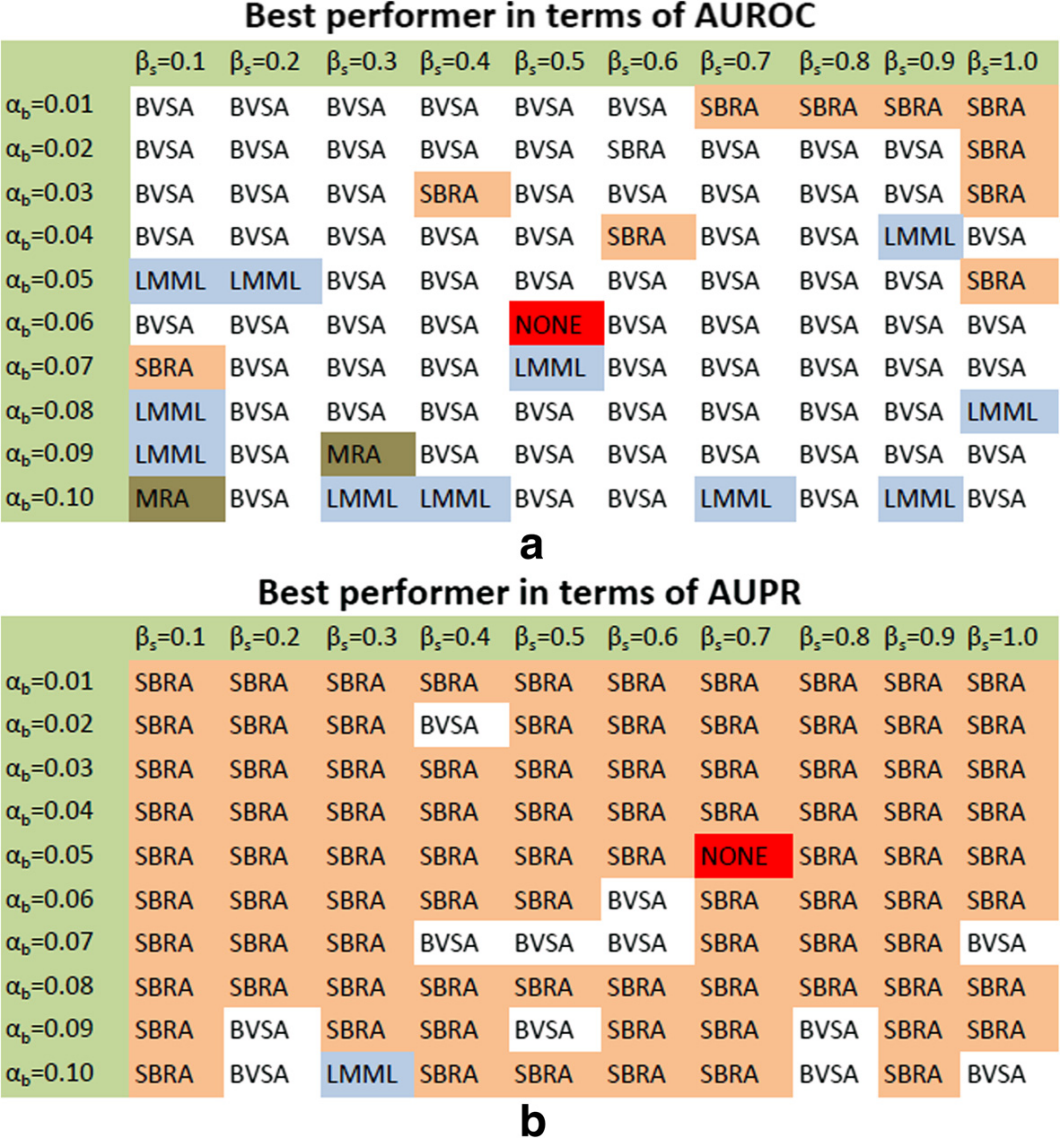
**Best performers in reconstructing the the MAPK pathway at different levels of signal dependent and independent noises.** The performances were evaluated for different levels of signal dependent (*β*_*s*_, *X*-axis) and independent (*α*_*b*_, *Y*-axis) components of the measurement errors. *β*_*s*_ and *α*_*b*_ were gradually increased from a minimum of 0.1 up to 1 and from 0.01 up to 0.1 respectively. The best performers in terms of maximum average AUROC(*p*<0.05)and maximum average AUPR(p<0.05) are shown in panels **(a)** and **(b)** respectively.

As in the case of BVSA, the performances of stochastic MRA, SBRA and LMML were also evaluated by generating 10000 datasets for each noise level (i.e. for each combination of *α*_*b*_ and *β*_*s*_ values) and executing these algorithms on each of these data sets. The resulting connection coefficient matrices (in case of stochastic MRA and LMML) and weight matrices (in case of SBRA) were then used to calculate the corresponding AUROC and AUPR values. The resulting AUROC and AUPR values (see Additional file 6: Table S1 and Additional file 7: Table S2) were compared with those calculated from the networks inferred by BVSA and two best performers, one with maximum average AUROC and one with maximum average AUPR, were selected (with p <0.05 ^1^) at each noise level (Figure 3). Our analysis reveals that, BVSA has the highest average AUROC in most of the cases, except a few sporadic cases where the other algorithms performed better (Figure 3(a)). By contrast, SBRA has the highest average AUPR in most of the cases (Figure 3(b)). This suggests that BVSA infers a larger number of interactions with reasonable accuracy, whereas SBRA infers a smaller number of interactions with relatively higher precision.

##### Network reconstruction from incomplete sets of perturbations

For real biological networks,it often is impossible to perturb each network module, separately or in combination. Accordingly, the resulting datasets usually do not contain complete information for a full reconstruction of the underlying network. Here we demonstrate that even in such cases BVSA can reveal salient features of network structures with better accuracy than its counterparts.

**Figure 4:**
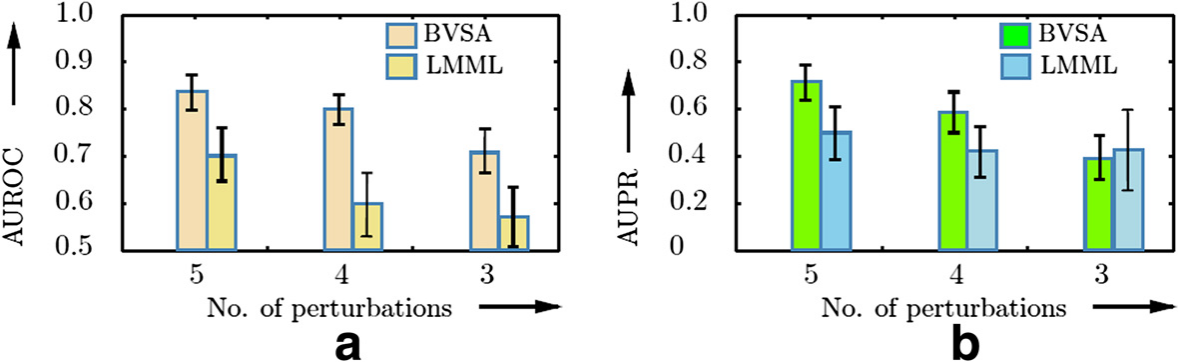
**Network reconstruction from incomplete perturbation data.***X*-axis represents the number of perturbations and *Y*-axis represents AUROC in panel **(a)** and AUPR in panel**(b)**. The error-bars indicate the standard deviations of the corresponding AUROCs in panel **(a)** and AUPRs in panel **(b)**. The results suggest that the networks reconstructed by BVSA is significantly more accurate than both LMML and random guesses even when only half of the MAPK modules were perturbed and no repetitions of the experiments were available.

Firstly, we simulated steady state responses of the MAPK pathway after perturbing only five out of six modules (adapter, initiator, MAP3K, MAP2K and MAPK) modules by knocking down Shc, Ras, Raf, MEK and ERK one at a time. We assumed that the knockdowns were performed with 80% efficiency. The simulations were performed stochastically to account for biological noise. Additionally, simulated measurement errors (*α*_*b*_ = 0.01, *β*_*s*_ = 0.1) were added to the perturbation responses. No repetitions of the knockdown experiments were performed. This yielded noisy steady state responses of the MAPK modules to five different perturbations. Classical MRA [19], its stochastic counterpart [16,17] and SBRA are unable to reconstruct a network from this dataset due to its rank deficiency. However, BVSA and LMML are designed to reconstruct networks in situations where the number of perturbation experiments is less than the number of network modules. We generated 10000 datsets with five perturbations (as described above) and inferred network structures from each of these datasets using BVSA and LMML. We then calculated average AUROC and AUPR values for each of the inferred networks. The AUROCs and AUPR values, calculated from the networks inferred by BVSA algorithm were then compared with those of the LMML algorithm to determine the best performer (see Figure 4). The procedure was repeated by perturbing only four (RasGDP, Raf, MEK, and ERK knockdowns) and three (Raf, MEK and ERK knockdowns) modules out of six. This analysis revealed that the performance of BVSA was significantly better than that of the LMML algorithm when faced with incomplete perturbation data.

In the simulation study of the MAPK pathway we established that BVSA can accurately infer network structures from perturbation data and it is robust against biological noises, measurement errors, and insufficient perturbation experiments. However, the above study does not demonstrate the scalability of BVSA, i.e. whether BVSA can be efficiently implemented to infer larger networks, e.g. GRNs consisting of hundreds or even thousands of genes. Below, we address this issue by using simulated perturbation responses of a 10 gene and a 100 gene GRN and compare its performance with that of (stochastic) MRA, SBRA and LMML.

##### Simulation study: in-silico GRNs

For this study we chose two in-silico gene regulatory networks which were previously provided as a part of the fourth network inference challenge of the DREAM consortium http://wiki.c2b2.columbia.edu/dream/index.php/ Challenges. The chosen networks are indexed as network 1 in the 10 gene and 100 gene categories, respectively, in the DREAM-4 data repository (http://wiki.c2b2.columbia.edu/dream/index.php/Challenges). The networks were perturbed by knocking out the component genes one by one. Following each perturbation the responses of the other genes in the network were measured. The knockout experiments were simulated using the GeneNetWeaver [30] software. No biological or technical replicates were simulated for the perturbation experiments. We used the normalized perturbation (knock out) responses for network inference.

We used BVSA, stochastic MRA [16], SBRA [7] and LMML [18] to infer the topologies of the above networks from the perturbation data provided by the DREAM consortium. In case of stochastic MRA, the connection coefficients were inferred using the TLSR algorithm [16], but the uncertainties surrounding the estimated values of the connection coefficients could not be inferred due to the lack of replicate experiments (see [17]). We executed each algorithm 50 times ^2^ on the same datasets and calculated: (a) the average AUROC and the corresponding standard deviation, (b) the average AUPR and the corresponding standard deviation, (c) the average time taken to finish execution for each of the four algorithms. The results of this analysis, along with the performances of the winning algorithms ([35,36]) in the 10 and 100 gene categories of the fourth DREAM challenge is shown in Table 1.

**Table 1:** **Performance comparison of BVSA, (stochastic)MRA, SBRA and LMML algorithms along with the winners in the 10 and 100 gene categories (**[35]**,**[36]**) of the DREAM4 challenge**

**Algorithm**	**10 Gene network**			**100 Gene network**		
	**AUROC**	**AUPR**	**Time (secs)**	**AUROC**	**AUPR**	**Time (secs)**
BVSA	0.9323 ± 0.0121	0.7311 ± 0.011	6.023 ± 0.119	0.85 ± 0.0101	0.14 ± 0.0108	1384.92 ± 12.8
stochastic MRA	0.9231	0.7133	0.0008	0.709	0.037	0.68
SBRA	0.7572 ± 0.019	0.58 ± 0.02	0.11 ± 0.02	0.65 ±0.003	0.075 ±0.01	1520 ± 3.319
LMML	0.8035 ± 0.06	0.66 ± 0.07	27.32 ± 1.73	0.644 ±0.02	0.04 ±0.001	41562 ± 3722.2
Kuffer et. al.[36]	0.972	0.916	NA	NA	NA	NA
Pinna et. al. [35]	0.764	0.590	NA	0.914	0.536	NA

The results are shown in mean ± std format. The information regarding the performance of Kuffner et. al.’s algorithm on the 100 gene dataset is not available since they did not participate in the 100 gene category of the DREAM4 challenge. The execution times of Pinna et. al.’s amd Kuffer et. al.’s algorithms were not published and therefore not available. Unavailble information is shown by ‘NA’ in the table.

The results (Table 1) suggest that in the 10 genes category BVSA outperformed most of the other algorithms (stochastic MRA, SBRA, LMML and that proposed by Pianna et. al. [35]) except that of Kuffner et. al. ([36], the winner of the DREAM4 in the same category) in terms of accuracy. A possible reason behind the fact that Kuffner et. al.’s algorithm performed better than BVSA is that their algorithm uses five different types of data, i.e. knockdown, time series, multi-factorial and double-knockout data in addition to the single knockout data for network reconstruction [36], whereas BVSA uses only single knockout dataset. The heterogeneous datasets provide a wealth of additional information about the network topology which BVSA is currently unable to use and therefore does not perform as well as Kuffner et. al.’s algorithm. In terms of execution time, BVSA took more time (on an average ≈ 6 seconds) to finish execution than SBRA (on an average ≈ 0.11 seconds) but less time than LMML (on an average ≈ 27.32 seconds) in the 10 gene category. The execution time of Kuffner et. al.’s algorithm is unavailable.

In the 100 genes category, BVSA outperformed most of the other algorithms (Stochastic MRA, SBRA and LMML) except that proposed by Pinna et. al. ([35], winner of DREAM4 challenge in the same category) in terms of accuracy. Kuffner et. al. did not participate in the 100 genes category. In terms of execution time, BVSA (on an average 23 minutes 5 seconds) outperformed both SBRA (on an average 25 minutes 20 seconds) and LMML (on an average 11 hours 32 minutes 47 seconds) in the 100 genes category. The execution time of Pinna et. al’s algorithm [35] is not available. In both 10 and 100 genes category stochastic MRA was the fastest with execution time of (on an average) ≈ 0.0008 seconds and ≈ 0.64 seconds respectively. This is due to the fact that we could not perform MCMC simulation for stochastic MRA to estimate the probability distributions of the connection coefficients. Instead, we calculated point estimates of the connection coefficients using the TLSR method [16]. However, if a MCMC simulation was performed, then the performance of the stochastic MCMC algorithm would have been considerably slower. This is demonstrated in the next section, where we used real biological data with multiple biological and technical replicates.

Encouraged by the above results we used BVSA to infer the topology of the ERBB regulated G1-S transition pathway in breast cancer cells from real experimental data.

##### Real datasets: ERBB regulated G1/S transition in human breast cancer cells

ERBB receptors are a family of four structurally related receptor tyrosine kinases (RTK) which form homodimers, heterodimers, and possibly higher-order oligomers upon activation by growth factors such as EGF, TGF- *α* etc. Activated ERBB dimers act as docking sites for a myriad of adapter proteins which simultaneously initiate many signaling cascades such as the AKT pathway, MAPK cascades, the JAK/STAT pathway etc. Many of these pathways tightly regulate different phases of cell cycle in eukaryotic cells.

At the end of G1 phase of cell cycle when the cells reach their final stage of growth they decide whether to divide, delay division or enter a resting stage. The decision making process involves phosphorylation of the retinoblastoma protein pRB by different Cyclin/CDK complexes. Unphosphorylated pRB proteins bind to E2F family of transcription factors and inhibit its activity. Upon phosphorylation, pRB proteins dissociate from E2F resulting in its activation. A eukaryotic cell commits to divide and initiates DNA replication (i.e. enter into S phase) when active E2F triggers transcription of the necessary genes. The ERBB regulated signaling pathways influence this mechanism by releasing Cyclin/CDK complexes from their inhibitor proteins (Cyclin Dependent Kinase inhibitors) p21 and p27. In 20-30% of breast cancers, ERBB2, a member of the ERBB family of receptors, is overexpressed resulting in a malfunction of control points in the cell division process and unrestricted growth. These cancers are usually treated with Trastuzumab, a recombinant antibody designed to block the ERBB2 activity. However, about two third of the ERBB2 overexpressing breast cancer patients are found to be Trastuzumab resistant ab. initio [37]. In these patients, the cancer cells are able to overcome the cell cycle arrest mechanisms even though ERBB2 is blocked by Trastuzumab. The mechanisms which allow the breast cancer cells to bypass cell cycle arrest is not well understood and currently under intense research.

**Figure 5:**
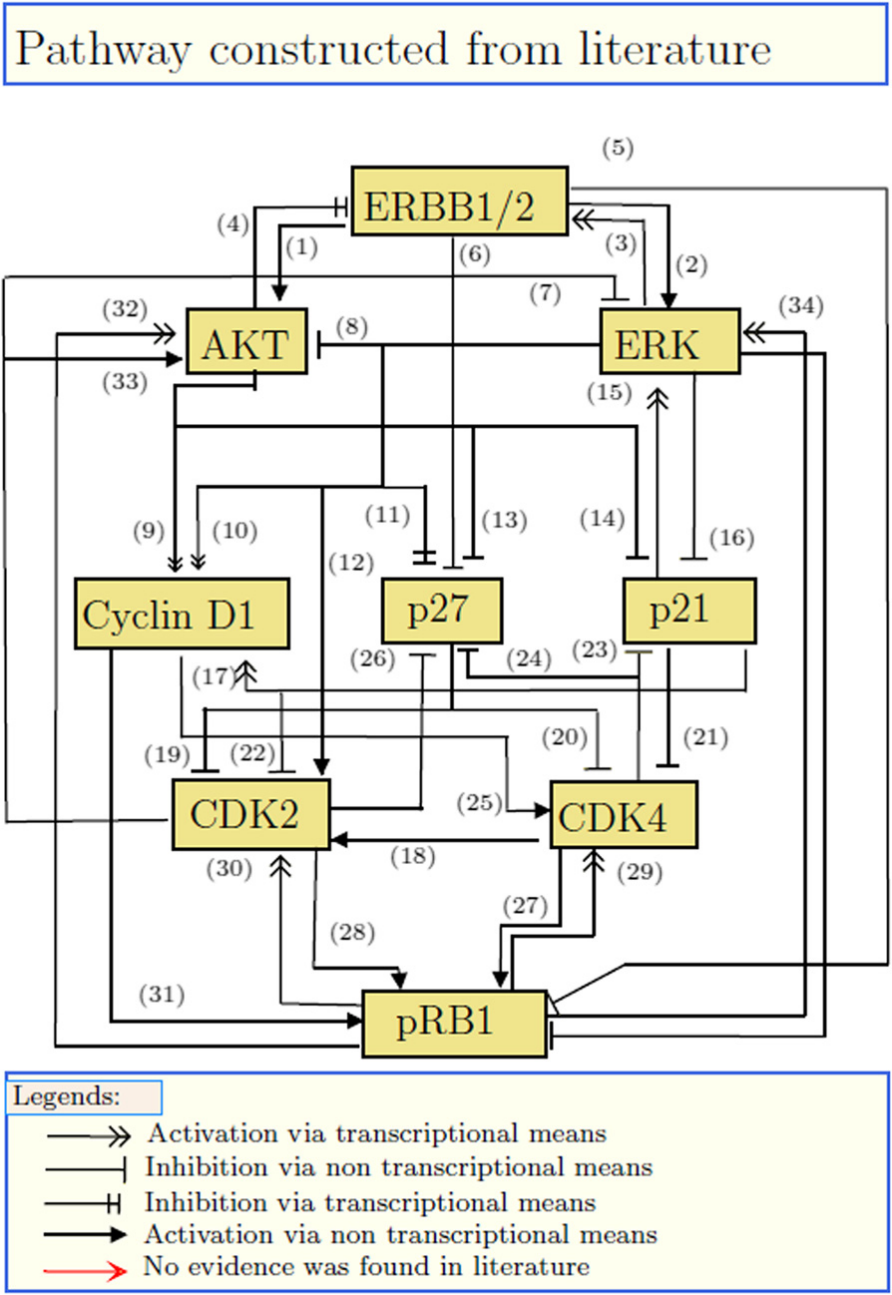
**A network of ERBB regulated G1/S transition constructed from the literature.** The numbers associated with the network edges point to corresponding references listed in the Additional file 8.

In a notable effort, Sahin et. al. systematically perturbed key components of ERBB mediated signaling pathways and the G1/S transition mechanisms in Trastuzumab resistant breast cancer cells to understand how the former influence the later and vice versa [37]. RNAi was used to individually knock down the expression of the genes corresponding to ERBB1, ERBB2, ERBB3, AKT, MEK, cMyc ER- *α*, IGF1R, p21, p27, CDK2, CDK4, Cyclin-D1, Cyclin E1 and pRB1 in HCC1954 cells [37]. The first seven of these proteins are part of the ERBB mediated signaling pathways and the rest are part of the G1/S transition mechanism. After each knockdown, the cells were stimulated with EGF for 12 hours and the expression levels of ERBB1,ERBB2, p21, p27, CDK2, CDK4, Cyclin-D1 and phosphorylation levels of ERK, AKT, pRB were measured using reverse phase protein arrays [37]. We analyzed these measurements^**3**^ using BVSA, (stochastic) MRA, SBRA and LMML to unravel the interactions among the above proteins. To estimate the accuracy of each of these algorithms, we first developed a literature based reference pathway (Figure 5) which represents our current knowledge about how the above proteins interact with each other to regulate G1/S transition in an ERBB dependent manner. Then we compared the topology of the reference pathway with those reconstructed by BVSA, MRA, SBRA and LMML. Below we describe the results of our analysis.

In case of BVSA, we used five parallel Gibbs samplers to search for the potential regulators of each protein. Each sampler was allowed to sample for 2000 iterations. The entire simulation took ≈3 minutes to complete on an intel core i7-820m processor based laptop computer with 12 Giga bytes of RAM. To see whether all parallel samplers converge to the same distribution we plotted (Additional file 9: Figure S3) the log-marginal log(*P*(***A***_*i*_|***R***)) of the samples drawn by the samplers. The parallel samplers converged rapidly to the same distribution. As before, we rejected 20% of the early samples as burn ins and the rest of the samples were used to calculate the posterior edge probabilities *P*_*ij*_. The posterior edge probabilities were then thresholded using the thresholding scheme described above.

**Figure 6:**
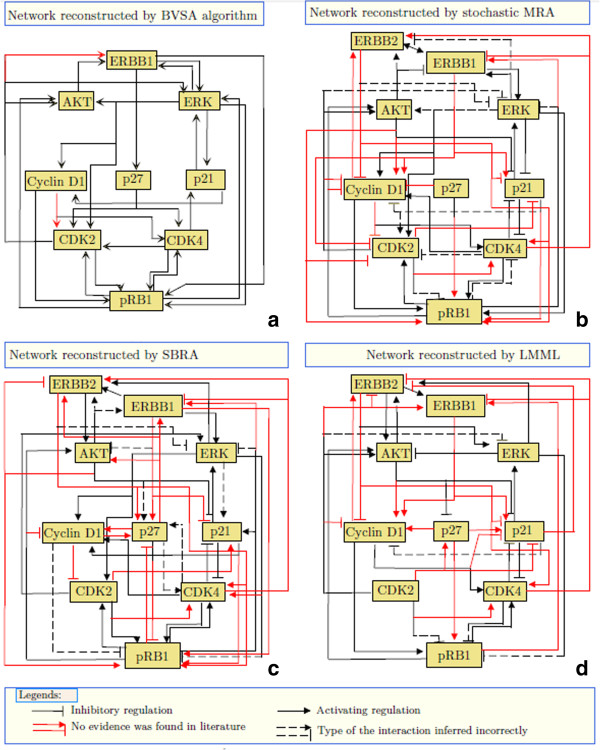
Reconstruction of the ERBB regulated G1/S transition mechanism by BVSA (panel a), stochastic MRA (panel b), SBRA (panel c) and LMML (panel d).

The inferred network (Figure 6(a)) reveals many well known mechanisms by which the ERBB mediated signaling pathways regulates the G1/S transition point of cell cycle. For example, the regulation of the CDK inhibitors p21 and p27 by the ERK pathway, the interplay between Cyclin-CDK complexes, their inhibitors (p21, p27) and their target protein pRB were identified. Some less recognized mechanisms of cell cycle regulation were also detected. For instance, p27 and pRB1 were found to be directly regulated by ERBB. It was previously demonstrated that Src and the JNK pathway, which are downstream to ERBB [38], can regulate the activity of p27 and pRB1 respectively in an AKT and ERK independent manner [39,40]. Since neither Src nor the components of the JNK pathway were measured in the perturbation experiments [37], the ERBB mediated regulation of p27 and pRB1 via these pathways were detected by BVSA as direct interactions.

Similarly, ERK was also found to directly regulate the activity of pRB1 (Figure 6(a)). Previous experimental results indicated that the activity of pRB1 can be regulated by the p53/MDM2 pathway [41] which itself is regulated by the ERK pathway [42]. Since p53 and MDM2 were not measured in the perturbation experiments [37], the ERK mediated regulation of pRB1 via this pathway was inferred as a direct interaction (Figure 6(a)).

We also identified a number of potential feedback mechanisms. For instance, pRB was found to feedback into its upstream kinases CDK2, CDK4 and even further upstream, into the kinases AKT and ERK (Figure 6(a)). Experimental studies by other researchers suggest that these feedback loops are mediated by the transcription factor E2F which is activated upon phosphorylation of pRB. Activated E2F directly binds to the CDK2 [43] promoter and activates its transcription. E2F is also found to transcriptionally regulate AKT1 [44] resulting in a feedback regulation of pRB. On the other hand, E2F transcriptionally activates PAC1 which dephosphorylates ERK [45] thereby completing a negative feedback loop. E2F also can activate ARF which upregulates the stability of the p53 protein [41]. p53 inhibits the translation of the CDK4 protein [46] forming a feedback loop.

Some of the feedback mechanisms identified in this analysis can potentially explain the observed Trastuzumab resistance in HCC1954 cells. In fact, our reconstructed model identified two feedback mechanisms which were experimentally proved by other researchers to cause Trastuzumab resistance in ERBB2 overexpressing breast cancer cells. These feedback loops involve AKT and ERK mediated regulation of ERBB receptors (Figure 6(a)). Previously, it was demonstrated that AKT, a downstream kinase of ERBB, inhibits ADAM17 which activates TGF- *α*, a potent ligand for ERBB receptors [47]. Inhibiting ERBB2 using Tratuzumab inhibits AKT and upregulates ADAM17 [47]. ADAM17 activates many ERBB ligands which keeps ERBB pathways activated [47]. However, the activity of ADAM17 was not measured in the perturbation experiments [37] which we considered for our analysis and the feedback regulation of ERBB by AKT via ADAM17 was inferred by BVSA as a direct network connection from AKT to ERBB.

Additionally, the ERK-ERBB feedback loop which was also inferred by BVSA as a direct feedback from ERK to ERBB is in-fact mediated by EGR1 [48], a target gene of the ERK pathway [49].

We found credible evidence in the literature to support all but two interactions inferred by BVSA. The literature references regarding the inferred interactions are provided in the SI. At the same time, a few known mechanisms involving ERBB regulated signaling pathways and the G1/S checkpoints were not identified by BVSA. In Figure 6(a), we have shown the identified, unidentified and falsely identified interactions.

We also used the Median Probability Model, i.e. *p*_*th*_ = 0.5 [50], to reconstruct the above pathway from the probability matrix ***P*** which was inferred by BVSA. The resulting network is shown in Additional file 10: Figure S4. The inferred network shares a number of interactions with that derived by the thresholding scheme which was proposed in this paper. However, it fails to identify some well known interactions which were successfully inferred by our proposed thresholding scheme, e.g. ERBB mediated regulation of ERK, the roles of Cyclin Dependent Kinase inhibitors, pRB1 mediated feedback regulations, the autocrine loops etc.

For further comparisons, we employed MRA [17]**], SBRA [**[7] and LMML [18] to reconstruct the ERBB2 regulated G1/S transition network from the same dataset as above [37]. In case of MRA, 10^**6**^ random realizations of the steady state perturbation responses were drawn from Gaussian distributions with means and standard deviations obtained from experimental data [17]. The connection coefficients were calculated from each realization of the perturbation responses using TLSR [16]. The resulting 10^**6**^ realizations of each connection coefficient *r*_*ij*_ were used to infer the structure of the ERBB regulated G1/S transition mechanism. In most cases, a few realizations (typically <1*%*) of a connection coefficient *r*_*ij*_ had very different values from the bulk of its values. These “outliers” were discarded by rejecting 1*%* extreme values of each *r*_*ij*_. The connection coefficients which had high variances even after rejecting the outliers were assumed to be unidentifiable and were discarded from the analysis. The values of the remaining connection coefficients were then subjected to a Z-test which calculates a p-value to determine whether its mean is close enough to 0. If the p-value is less than 0.05 then the mean of the *r*_*ij*_ is significantly different from 0, i.e. in this case, *r*_*ij*_ represents a true network connection. We then used the Benjamini-Hochberg [51] procedure to correct for multiple testing and eliminate any falsely discovered network connection. To determine whether *r*_*ij*_represents an activating or inhibitory interaction we first calculated the histogram of each *r*_*ij*_. The histograms are shown in Additional file 11: Figure S5. If the fraction of negative realizations of *r*_*ij*_is larger than the fraction of positive realizations then *r*_*ij*_ is assumed to represent an inhibitory interaction. Otherwise, it represents an activating interaction. The above procedure took approximately 3 hours and 27 minutes to complete by the same computer which was used to implement BVSA on the ERBB2 dataset. The network which was reconstructed this way is shown in Figure 6(b). Stochastic MRA inferred many well known interactions (represented by black lines in Figure 6(b)) which take part in the ERBB2 mediated G1/S transition control mechanism. However, it also inferred a large number of interactions (represented by red lines in Figure 6(b)) which could not be supported by evidence from the literature. These interactions are most probably falsely identified interactions.

Furthermore, we reconstructed the same pathway using SBRA. SBRA does not infer connection coefficients. Instead, it infers a weight matrix ***W*** which represents the strength of the interactions. The sign of the elements of ***W*** represents whether the corresponding interaction is activating or inhibitory. SBRA took approximately 1 minute and 20 seconds to execute as opposed to 3 minutes for BVSA and 3 hours 20 minutes for MRA. The network structure constructed from the inferred weight matrix is shown in Figure 6(c). Similar to MRA, SBRA also inferred a number of well known interactions along with a large number of interactions which are most likely to be false positives.

Finally, we reconstructed the ERBB pathway using LMML [18]. It took approximately 35 minutes and 27 seconds to finish executaion as opposed to 3 minutes for BVSA, 1 minutes 20 seconds for SBRA and 3 hours 20 minutes for MRA. The network inferred by LMML is shown in Figure 6(d). LMML also inferred many known interactions along with a relatively large number of interactions which could not be supported by literature evidence.

The above analysis suggests that BVSA provides an overall faster and more accurate solution to the network reconstruction problem when compared to other network inference algorithms such as MRA, SBRA and LMML. However, our comparison of accuracy depends on the reference ERBB pathway which was constructed from literature. We selected only highly cited experimental results to construct the reference pathway. However, not all of these experiments were performed on the same cell line as the one used by Sahin and colleagues [37]. Therefore, the reference pathway (Figure 5) should only be treated as a plausible generic mechanism of ERBB mediated G1/S transition and the result of the comparative analysis presented in this section should be treated with its fair share of scepticism.

## Discussion and conclusion

In this paper, we propose a network inference algorithm which combines modular response analysis with Bayesian variable selection techniques. This algorithm is capable of reconstructing network topologies from noisy perturbation responses of biochemical systems. It is more accurate than two previously proposed stochastic formulations of MRA, one based on TLS regression [16] and the other based on repeated TLS regressions using an MCMC sampler [17]. The increased accuracy of BVSA is a result of the fact that BVSA penalizes dense networks by implementing appropriate prior distributions for the unknown variables (e.g. *r*_*ij*_, *A*_*ij*_), thereby minimizing the possibilities of false positives, whereas the stochastic MRA methods lack this capability due to lack of appropriate regularization techniques. The proposed BVSA algorithm is also performs better than a recently proposed Levenberg-Marquardt optimization based Maximum Likelihood (LMML) method [18] and a previously developed sparse Bayesian regression method (SBRA) [7]. This is most likely due to the fact that BVSA implements a model averaging technique, which determines the network topology by averaging a set of likely network models, whereas LMML and SBRA implement two different model selection techniques, each of which find a single network model that maximizes a likelihood function. It was shown by many researchers [52]**-**[54] that model averaging performs better than model selection (for a theoretical explanation see [53,55]) which may explain why BVSA performs better than LMML and SBRA. We also demonstrated that BVSA can reconstruct network topologies even when the number of perturbation experiments are not sufficient for a full network reconstruction using other algorithms such as MRA [14] and SBRA [7]. It is computationally less expensive compared to many other statistical network inference algorithms, e.g. MCMC based MRA [17], SBRA (for large networks) [7] and LMML [18]. However, the capability of the BVSA algorithm is limited to inferring binary interactions, whereas MRA, SBRA and LMML can also infer the connection coefficients which represent the strength and type (activating or inhibitory) of each interaction. Such information is necessary to understand the molecular mechanisms by which a biochemical network operates. Although, BVSA cannot directly estimate the connection coefficients, these quantities can be readily estimated using linear regression, once a binary network topology is inferred using BVSA algorithm. However, a more systematic approach in estimating the connection coefficients from perturbation data needs to be developed. Therefore, in our future research, we plan to extend the BVSA algorithm to infer the connection coefficients of biochemical networks.

Additionally, BVSA is vulnerable to collinearity in experimental data [56], i.e. if perturbation responses of different network nodes are collinear then BVSA may not perform to its full potential. Therefore, one must practice caution in designing the perturbation experiments and make sure that the perturbation responses of different network nodes are as orthogonal as possible.

The biggest concern of using statistical network inference algorithms to analyze biological datasets is the reliability of the predicted networks. One way of increasing reliability is to make systematic use of all existing information regarding the biochemical networks which the researcher wants to explore [3]. BVSA, at its current stage, incorporates only subjective knowledge regarding abstract topological properties of generic biochemical systems in its inference engine. To improve its accuracy and reliability, it should be customized to take network specific objective knowledge into account. In our future research, we plan to focus on incorporating network specific knowledge into the inferential framework of the BVSA algorithm and thereby increasing its accuracy.

## Methods

### The prior distributions of the unknown variables

#### The prior distribution of the binary variables *A*_*ij*_

Biochemical entities such as genes and proteins interact with only selective groups of partners, making biochemical networks sparse systems. Network sparsity implies that for any two arbitrary nodes *i* and *j*, *A*_*ij*_ has a small probability of being 1, typically *P*(*A*_*ij*_ = 1)<0.5 Therefore, if we denote *P*(*A*_*ij*_ = 1) = *θ* then *θ* indicates the sparsity of the network. The degree of sparsity of a biochemical network is usually unknown beforehand (a priori), implying that our knowledge surrounding the probable values of *θ* is uncertain. To formulate our uncertainty about *θ*, we assumed that it has a Beta distribution with parameters *a*, *b*. The choices of the values for *a* and *b* represent our prior knowledge about the sparsity of the network. If the network is likely to be sparse, which is a reasonable a priori assumption for biological networks, then we choose *a*>*b*, since, intuitively *a* and *b* represent our prior knowledge about the likely frequencies of 1’s and 0’s occuring in the binary adjacency matrix ***A***. By the same rationale, we choose *b*>*a* when the network is believed to be dense (*P*(*A*_*ij*_ = 1)>0.5). BVSA algorithms were shown to perform robustly for different values of *a* and *b*, if these values correctly represent the prior knowledge of model sparsity [57].

Following this notion, we assigned *a*=1 and *b*=2. These values imply that the probability of node *i* being regulated by an arbitrary node *j* is most likely but not limited to be within the range [ 0.097,0.57], i.e. 0.097≤*P*(*A*_*ij*_ = 1)≤0.57 (see Additional file 1 for explanation) which broadly represents our prior assumption that biochemical networks are sparse.

#### The prior distribution of the connection coefficients *r*_*ij*_

We conceptually divide a *n* node network into *n* number of smaller subnetworks, each of which corresponds to the interactions between a specific node (*i*) and its regulators, whose interactions with nodes other than *i* are not considered. Thus, each subnetwork (*i*) includes only node *i* and the nodes that directly affect node *i*, termed regulators of this node. These subnetworks can be treated as independent networks and their topologies can be inferred separately [58,59]. In this case, one only needs to account for the interdependence of the connection coefficients within each subnetwork. We assigned a ‘spike and slab’ [60] type joint probability distribution for the connection coefficients of each individual subnetwork. By definition, the *i*^*th*^subnetwork consists of the interactions between node *i* and its regulators, and the connection coefficients corresponding to these interactions are denoted by $r_{i}=\{r_{\text{ij}};j=1,\ldots ,n;j\neq i\}$. The elements of ***r***_*i*_ which do not represent true edges are considered to be 0 with probability 1 (the spikes) and the elements which represent true edges (denoted by ***ρ***_*i*_) are assumed to have a multivariate Gaussian distribution (the slab) with mean ***0*** and covariance matrix $V_{\rho_{i}}$. Assuming that ***ρ***_*i*_ has $n_{k}^{i}$ elements, $V_{\rho_{i}}$ is a $n_{k}^{i}\times n_{k}^{i}$ matrix which represents our prior knowledge about the possible range of values of ***ρ***_*i*_ while accounting for the dependencies among different elements of ***ρ***_*i*_. A commonly used approach is to assume that the prior covariance matrix $V_{\rho_{i}}$ is proportional to the posterior covariance matrix, i.e. $V_{\rho_{i}}\propto \sigma^{2}{\left(R_{\text{pr}(i)}R_{\text{pr}(i)}^{T}\right)}^{-1}$[61] where ***R***_*pr*(*i*)_ is a $n_{k}^{i}\times n_{p}^{i}$ matrix whose rows represent the regulators of node *i* and the columns represent the global responses of the regulators to different perturbations. If $n_{p}^{i}<n_{k}^{i}$ i.e., the number of perturbations are less than the number of regulators of node *i* then the matrix $\left(R_{\text{pr}(i)}R_{\text{pr}(i)}^{T}\right)$ is not invertible and therefore, $V_{\rho_{i}}$ becomes a singular matrix. In such scenarios, the posterior distribution of the binary variable *A*_*ij*_does not exist. One way to ensure positive-definiteness of $V_{\rho_{i}}$ is to introduce a ridge parameter (*λ*) in its formulation [62]. The resultant $V_{\rho_{i}}$ is shown below.

(6) $$V_{\rho_{i}}=c\sigma^{2}{\left(R_{\text{pr}(i)}R_{\text{pr}(i)}^{T}+\mathrm{\lambda I}\right)}^{-1}$$

In Eq. 6, *c* is the proportionality constant which represents how much importance is attributed to the prior precision^**4**^$V_{\rho_{i}}^{-1}$. The performances of variable selection algorithms such as ours are sensitive to the value of the parameter *c*[63]. Several intuitive choices for the values of *c*, their implications and effects on the performances of these algorithms are discussed in detail in [63]. Some alternatives to these popular choices had also been proposed previously. For example, George et. al. [64] and Hansen et. al. [65] proposed to estimate the likely values of *c* from data using empirical Bayes techniques. However, this was criticized on the grounds that empirical Bayes methods do not correspond to solutions based on Bayesian or formal Bayesian procedures. Liang et. al [63] proposed a full Bayesian solution to the above problem, but this solution involves calculating hyper-geometric distributions which becomes computationally highly expensive. Hence, we assigned a simple, computationally inexpensive value $c=n_{p}^{i}$ drawing on the notion that the amount of information contained in the prior equalize the amount of information in one observation. It was shown that the adopted value performs well for most scenarios except for cases where a very large number of replicate datasets are available [29]. However, such a scenario is unlikely to occur in biological experiments, where the contrary problem of having fewer replicates than wanted is more frequently encountered.

The value of *λ* was arbitrarily chosen to be 0.1 since it was previously shown that any reasonable value within the range 0<*λ*<1 works equally well[62] in most cases. The introduction of the ridge parameter in $V_{\rho_{i}}$ ensures the existence of the posterior distributions of *A*_*ij*_ even when a network has far more nodes than the number of perturbations performed.

##### The prior distribution of the error *∊*_*ik*_

*∊*_*ik*_ is a linear combination of the noise present in individual measurements [66]. Therefore, by the central limit theorem, *∊*_*ik*_ is a Gaussian random variable [66,67]. We assumed that *∊*_*ik*_ is equally likely to have positive or negative values and hence its distribution is centered around 0, i.e. has zero mean. The variance (*σ*^**2**^) of *∊*_*ik*_ depends on biological noises and measurement errors and can vary drastically depending on the type of network being investigated and measurement systems used in the investigation. Therefore, our knowledge about the true nature of the noise variance *σ*^**2**^ is uncertain. To account for the uncertainties in the noise variance *σ*^**2**^, we assumed that *σ*^**2**^ has an inverse gamma distribution with scale parameter *α* and location parameter *β*. The values of *α* and *β* are chosen to incorporate any prior knowledge about the noise variance into the formulation. In the absence of such knowledge, one may choose values for *α* and *β* which yield flat and non-informative priors for *σ*^**2**^. Following this notion, we selected *α*=1 and *β*=1 to ensure that *σ*^**2**^ has a flat prior which implies that it can have a wide range of positive values.

### The posterior distribution of the binary variable *A*_*ij*_

The posterior distribution of the binary variables corresponding to each subnetwork was calculated separately. Let us denote by ***A***_*i*_, the binary variables corresponding to the subnetwork which consists of the interactions between node *i* and its regulators. The joint posterior distribution of its elements $\left\{A_{\text{ij}},j=1,\ldots ,n;j\neq i\right\}$ is shown below.

(7) p(Ai|R)∝npi−nki2|Rpr(i)Rpr(i)T|12|Vρi−1+Rpr(i)Rpr(i)T|12b1(n2+1)(n−1)nkiBeta(α+nki,β+n−nki−1)where,b1=1+0.5(RiRiT−RiRpr(i)T×(Vρi−1+Rpr(i)Rpr(i)T)−1Rpr(i)RiT)

Step by step analytical calculations which lead to the above expression are illustrated in Figure 1 and described in detail in the Additional file 1. However, Eq. 7 allows one to calculate the posterior probability of ***A***_*i*_ only up to a constant of proportionality.

To determine the true posterior of ***A***_*i*_ one needs to calculate the proportionality constant for Eq. 7 which requires the calculation of the right hand side of Eq. 7 for all possible configurations of ***A***_*i*_. Since, the elements of ***A***_*i*_ can be either 1 or 0, there can be 2^*n*−1^ possible configurations of ***A***_*i*_. For small networks (typically *n*<20) it is possible to exhaustively calculate the proportionality constant. In case of large networks (typically *n*≥20) exhaustive enumerations of Eq. 7 for all possible configurations of ***A***_*i*_ are prohibitively time consuming. In such cases one needs to approximate the posterior of ***A***_*i*_ using MCMC sampling.

### Approximating the posterior distribution of *A*_*ij*_ using Gibbs sampling

We implemented a Gibbs sampler for approximating the posterior distribution of ***A***_*i*_. The Gibbs sampler starts with a random realization of ***A***_*i*_($A_{i}^{0}$) and generates a sequence of samples $A_{i}^{1},A_{i}^{2},\ldots A_{i}^{N_{T_{s}}}$, where $N_{T_{s}}$ is the number of samples generated by the sampler. The *t*^*th*^ sample $A_{i}^{t}$ is obtained componentwise by sampling consecutively from the conditional distributions

(8) $$A_{\text{ij}}^{t}∼P(A_{\text{ij}}^{t}|\{A_{i1}^{t},A_{i2}^{t},\ldots A_{i(j-1)}^{t},A_{i(j+1)}^{t-1},\ldots A_{\text{in}}^{t-1}\},R)$$

for all *j*≠*i*. Each distribution shown in Eq. 8 is a Bernoulli with probabilities:

(9) $$\begin{array}{lll} & \;P(A_{\text{ij}}^{t}=1|\{A_{i1}^{t},A_{i2}^{t},\ldots A_{i(j-1)}^{t},A_{i(j+1)}^{t-1},\ldots A_{\text{in}}^{t-1}\},R)=\frac{p_{1}}{p_{1}+p_{0}}\;\\ & \;P(A_{\text{ij}}^{t}=0|\{A_{i1}^{t},A_{i2}^{t},\ldots A_{i(j-1)}^{t},A_{i(j+1)}^{t-1},\ldots A_{\text{in}}^{t-1}\},R)=\frac{p_{0}}{p_{1}+p_{0}}\; & \;\\ & \;\text{where,}p_{1}=P(\{A_{i1}^{t},A_{i2}^{t},\ldots A_{i(j-1)}^{t},1,A_{i(j+1)}^{t-1},\ldots A_{\text{in}}^{t-1}\}|R)\; & \;\\ & \text{and}p_{0}=P(\{A_{i1}^{t},A_{i2}^{t},\ldots A_{i(j-1)}^{t},0,A_{i(j+1)}^{t-1},\ldots A_{\text{in}}^{t-1}\}|R)\; & \; \end{array}$$

*p*_**1**_ and *p*_**0**_ in Eq. 9 can be calculated using Eq. 7.

Repeated successive sampling of Eq. 9 for all components of ***A***_*i*_ produces the sequence of samples $A_{i}^{t},t=1\ldots N_{T_{s}}$ which is a homogeneous ergodic Markov chain that converges to its unique stationary distribution *P*(***A***_*i*_|***R***). A practical consequence of this property is that as the length of the sequence is increased, the empirical distribution of the realized values of ***A***_*i*_ converges to the actual posterior *P*(***A***_*i*_|***R***). In our applications, we were not concerned about strict convergence of the Gibbs sampler. Instead, we adopted an approach similar to [68]**-**[70]. We initiated multiple parallel samplers each starting with a random configuration of ***A***_*i*_. Each sampler was allowed to generate a sequence of length $N_{T_{s}}$. We were satisfied if the parallel samplers showed broadly similar marginal distributions, i.e. they converged on each other. We rejected a number ($N_{T_{b}}$) of early samples from each of the sequences and assumed that the empirical distribution of the rest of the samples approximates *P*(***A***_***i***_|***R***). We have shown some illustrations of our approach in the results section.

The samples drawn after the “burn in” period can be used to calculate the posterior probability of *A*_*ij*_ = 1 which represents an individual edge emanating from node *j* to node *i*. An asymptotically valid estimate of the posterior probability (*P*_*ij*_) was calculated as shown below:

(10) $$P_{\text{ij}}=\frac{1}{N_{c}\times (N_{T_{s}}-N_{T_{b}})}\overset{N_{c}}{\underset{k=1}{\sum }}\overset{N_{T_{s}}}{\underset{t=N_{T_{b}}+1}{\sum }}A_{\text{ij}}^{\left(\text{tk}\right)}$$

Here, *N*_*c*_ is the number of Gibbs samplers initiated for each ***A***_*i*_.

### Thresholding the posterior probabilities of *A*_*ij*_

The topology of the underlying network can be determined by thresholding *P*_*ij*_ with a threshold probability *p*_*th*_, i.e., if *P*_*ij*_≥*p*_*th*_ it can be assumed that node *j* directly regulates node *i* and if *P*_*ij*_<*p*_*th*_then node *j* does not directly regulate node *i*. The value of *p*_*th*_ should be chosen carefully. During the performance evaluation phase, when the network topology is known, the standard approach is to construct a series of networks for different values of *p*_*th*_ in the range [ 0,1]. The topology of each network is then compared with the known topology and the overall performance of the algorithm is determined using Receiver Operating Characteristics (ROC) curves. This procedure is discussed in details in the results section.

When the network structure is unknown, determining the correct *p*_*th*_is crucial. In this case, the most commonly used approach is the Median Probability Model (MPM) [50] which simply assumes *p*_*th*_**=0.5. It has been shown that under certain conditions MPM ensures optimal performance [**[50]. However, when the data is highly collinear (which is almost always true in our case) choosing *p*_*th*_ = 0.5 no longer yields optimal results [71]. Therefore, we propose a simple and intuitive thresholding scheme which assumes that if an interaction occurs with higher than the average posterior edge probability then it is likely to be a true interaction, i.e. $p_{\text{th}}=\frac{1}{n(n-1)}\sum_{i=1}^{n}\sum_{j=1,j\neq i}^{n}P_{\text{ij}}$. Note that when *P*_*ij*_ is uniformly distributed within the interval [ 0,1], *p*_*th*_≈0.5 and our thresholding scheme resembles MPM. However, high level of multicollinearity often results in *P*_*ij*_<0.5 even when there is a direct influence from node *j* to node *i*[71]. In this case, as shown in the result section, our thresholding method outperforms MPM.

## Endnotes

^**1**^ Based on Benjamini-Hochberg corrected t-test between the AUROCs and AUPRs of the best and second best performers.

^**2**^ All computations were performed in a laptop computer equipped with core i7-3610Qm processor and 20 Gigabytes of Random access memory.

^**3**^ we considered only those perturbations which directly targeted the measured proteins. Only nine out of ten measured proteins were targeted by their corresponding siRNA. pRB was not targeted for siRNA mediated knockdown.

^**4**^ Precession is the inverse of variance.

## Competing interests

The authors declare that they have no competing interests.

## Authors’ contributions

TS performed the simulations and wrote the manuscript, WK and BNK designed the experiments and wrote the manuscript. All authors read and approved the final manuscript.

## Supplementary Material

Additional file 1 **Details of Bayesian formulation.** In this file we have described the mathematical details of the Bayesian formulation presented in the paper. Click here for file

Additional file 2 **source-code.** This file contains the MATLAB source code for the BVSA algorithm which is described in this paper. Click here for file

Additional file 3 **MAPK model.** In this file, we have provided the details of the ODE and the SDE models which were created to simulate the noise free and noisy perturbation response of the MAPK pathway respectively. Click here for file

Additional file 4 **Figure S1.** In this figure, we have illustrated the convergence of the Gibbs samplers which were created to reconstruct the MAPK pathway from noise free simulation data. Click here for file

Additional file 5 **Figure S2.** In this figure, we have illustrated the convergence of the Gibbs samplers which were created to reconstruct the MAPK pathway from noisy simulation data. Click here for file

Additional file 6 **Supplementary Table S1.** In this table we have shown the AUROCs and their standard deviations calculated from the MAPK pathway topologies reconstructed by BVSA, stochastic MRA, SBRA and LMML at different levels of signal dependent and independent noises. Click here for file

Additional file 7 **Supplementary Table S2.** In this table we have shown the AUPRs and their standard deviations calculated from the MAPK pathway topologies reconstructed by BVSA, stochastic MRA, SBRA and LMML at different levels of signal dependent and independent noises. Click here for file

Additional file 8 **References for ERBB pathway.** In this file we have provided the references for different interactions of the ERBB pathway. Click here for file

Additional file 9 **Figure S3.** In this figure, we have illustrated the convergence of the Gibbs samplers which were created to reconstruct the ERBB-G1/S transition pathway from experimentally obtained perturbation data. Click here for file

Additional file 10 **Figure S4.** In this figure, we have shown the topology of the ERBB-G1/S transition network as reconstructed by the Median Probability Model. Click here for file

Additional file 11 **Figure S5.** In this figure, we have shown the histograms of the connection coefficients of the ERBB regulated G1/S transition pathway as calculated by the stochastic MRA algorithm. Click here for file
