# Supplementary material for: Integrating Bayesian variable selection with Modular Response Analysis to infer biochemical network topology
Source: BMC Syst Biol. 2013 Jul 6;7:57. doi: 10.1186/1752-0509-7-57 (PMC3726398; doi:10.1186/1752-0509-7-57)
Supplement: Additional file 1 — Details of Bayesian formulation. In this file we have described the mathematical details of the Bayesian formulation presented in the paper. [file 1752-0509-7-57-S1.pdf]

Supplementary text: Details of the Bayesian formalisms

Tapesh Santra, Walter Kolch, Boris N. Kholodenko

April 13, 2013

The model: The global responses of a network node i (to perturbations which do not directly affect node i) have a linear relationship with the global responses of its potential regulators. This relationship was previously derived in [3] and the resulting formulation is known as Modular Response Analsys or MRA. We proposed a modified version of MRA by introducing a binary indicator variable A_{ij} which indicates whether node i is directly regulated by node j. Please note that any interaction routes via unknown elements are also considered as direct interaction. $A_{ij} = 1$ indicates that the node i is directly regulated by node j and $A_{ij} = 0$ indicates that node j does not directly regulate node i. We also accounted for biological and measurement noise in the MRA relationship. The resulting equation is shown below.

$$\sum_{j=1\dots n, j\neq i} A_{ij} r_{ij} R_{jk} + \epsilon_{ik} = R_{ik}; k = 1 \dots n_p^i \tag{1}$$

Here, r_{ij} are the connection coefficients (or local response coefficients[3]). R_{jk} is the global response of node j to perturbation k. ϵ_{ik} represents random noise. The global response coefficients (R_{ij}) are calculated from the perturbation responses. The binary variables A_{ij}, the connection coefficients r_{ij} and the noise ϵ_{ik} are unknown variables. Let us denote by $\boldsymbol{\rho}_i$ the set of connection coefficients which represent true network interactions directed to node i. The connection coefficients r_{ij} and the binary variables A_{ij} share an intricate relationship: if $A_{ij} = 1$ then $r_{ij} \in \boldsymbol{\rho}_i$ and if $A_{ij} = 0$ then $r_{ij} \notin \boldsymbol{\rho}_i$. Given these notations, Eq. 1 can be rewritten in a matrix form as shown below.

$$\boldsymbol{R}_i = \boldsymbol{R}_{pr(i)}^T \boldsymbol{\rho}_i + \boldsymbol{\epsilon}_i \tag{2}$$

Here, $\boldsymbol{R}_i = \left[R_{ik}, k = 1, 2, \dots n_p^i\right]^T$ is a $n_p^i \times 1$ vector which represents the global responses of node i and $\boldsymbol{R}_{pr(i)}$ is a $n_k^i \times n_p^i$ matrix which represents the global responses of the potential regulators of node i to perturbations $k = 1, 2, \dots n_p^i$. $\boldsymbol{\epsilon}_i = \left[\epsilon_{ik}, k = 1, 2, \dots n_p^i\right]^T$ is an $n_p^i \times 1$ vector that represents the cellular and measurement noise encountered in the perturbation experiments. Eq. 2 represents a model that describes how the potential regulators of node i regulates its perturbation responses. Inference can be drawn about different aspects of the model under a Bayesian framework. Bayesian inference of the model variables requires assigning prior distributions to the unknown variables of the model. The prior distributions allow us to incorporate our prior knowledge about the system. We have used generic priors for our framework which incorporates only subjective knowledge about biochemical networks rather than objective knowledge about particular networks that are being investigated. The prior distributions for the Bayesian framework is described below.

The priors: We start with the prior distribution of the binary vector $\boldsymbol{A}_i$ which represents the potential regulators of node i. The elements A_{ij} of the binary vector $\boldsymbol{A}_i = \{A_{ij}, j = 1 \dots n, j \neq i\}$ which represents the potential regulators of node i is assumed to have a Bernoulli distribution with parameter θ.

$$\begin{aligned} P(A_{ij} = 1|\theta) &= \theta \\ P(A_{ij} = 0|\theta) &= (1-\theta) \end{aligned} \tag{3}$$

Here θ is a parameter which represents the probability that node j directly regulates node i. If θ is known then the prior probability of $\boldsymbol{A}_i$ is given by:

$$P(\boldsymbol{A}_i|\theta) = \prod_{j=1, j\neq i}^{n} P(A_{ij}|\theta) = \theta^{n_k^i}(1-\theta)^{n-n_k^i-1} \tag{4}$$

where n is the total number of nodes in the network and n_k^i is the number of potential regulators of node i.

In most cases, the value of θ is not known a priori. However, it is well known that most biochemical networks are sparse implying that θ is most likely to be less than 0.5. Therefore we assumed that θ has a Beta-distribution with hyper parameters a and b, i.e.

$$\theta|a, b \sim Beta(a, b) \tag{5}$$

Here, we considered $a = 1$ and $b = 2$, which implies that $E(\theta|a,b) = \frac{a}{a+b} = 0.333$ and $\sigma = \sqrt{\frac{ab}{(a+b)^2(a+b+1)}} = 0.2357$, i.e. the values of θ is most likely, but not confined to be within the range $(0.0973, 0.568)$. When, θ itself is stochastic the marginal distribution of $\boldsymbol{A}_i$ can be estimated by marginalizing θ in Eq. 4, i.e.

$$P(\boldsymbol{A}_i|a,b) = \int_0^1 P(\boldsymbol{A}_i|\theta)P(\theta|a,b)d\theta \propto Beta(a + n_{k_i}, b + n - 1 - n_{k_i}) \tag{6}$$

Furthermore, there are $\binom{(n-1)}{n_k^i}$ possible different configurations of $\boldsymbol{A}_i$ which have n_k^i numbers of '1's. Therefore the probability that $\boldsymbol{A}_i$ has n_k^i number of '1's is

$$P(\sum \boldsymbol{A}_i = n_k^i|a,b) \propto \binom{(n-1)}{n_k^i} Beta(a + n_{k_i}, b + n - 1 - n_{k_i}) \tag{7}$$

For notational simplicity we shall denote $P(\sum \boldsymbol{A}_i = n_k^i|a,b)$ simply by $P(\boldsymbol{A}_i)$ hereafter.

For a network node i, the connection coefficients which represent true network connections ($\boldsymbol{\rho}_i$) directed to node i are assumed to have multivariate Gaussian distribution with zero mean and covariance matrix $\boldsymbol{V}_{\rho_i}$. A common practice is to assume that the prior covariance matrix $\boldsymbol{V}_{\rho_i}$ is proportional to the posterior covariance matrix that arises from experimental data [4, 1]. One way to estimate the posterior covariance matrix is to calculate its lower bound which is the inverse Fisher Information Matrix (FIM) of $\boldsymbol{\rho}_i$, denoted by $(\boldsymbol{R}_{pr(i)}\boldsymbol{R}_{pr(i)}^T)^{-1}$; where $\boldsymbol{R}_{pr(i)}$ contains the global responses of the potential regulators of node i to perturbations which do not directly affect node i. The resulting prior estimate of $\boldsymbol{V}_{\rho_i} = c\sigma^2(\boldsymbol{R}_{pr(i)}\boldsymbol{R}_{pr(i)}^T)^{-1}$ is known as Zellner's prior [4]. Here, the proportionality constant c represents how much importance is attributed to the prior precision $\boldsymbol{V}_{\rho_i}^{-1}$. Zellner's prior is widely used in Bayesian variable selection and model averaging frameworks. However, the invertibility of FIM depends on the rank of $\boldsymbol{R}_{pr(i)}$. Since, n_k^i is the number of potential regulators of node i and n_p^i is the number of perturbation experiments that do not directly affect node i, the dimension of $\boldsymbol{R}_{pr(i)}$ is $n_k^i \times n_p^i$. In order for FIM to be invertible, n_k^i must be less than or equal to n_p^i, i.e. one need to perform at least as many perturbation experiments as there are potential regulators of node i. When the FIM is not invertible, the Zellner's g prior becomes singular[1]. To guarantee identifiability of $\boldsymbol{\rho}_i$ in its posterior distribution one needs to ensure that $\boldsymbol{\rho}_i$ has a non singular distribution. One way to ensure a nonsingular prior distribution of $\boldsymbol{\rho}_i$ is to introduce a ridge parameter λ in the prior estimate of $\boldsymbol{V}_{\boldsymbol{\rho}_i}$ as shown below [2]:

$$\boldsymbol{V}_{\rho_i} = c\sigma^2(\boldsymbol{R}_{pr(i)}\boldsymbol{R}_{pr(i)}^T + \lambda I)^{-1} \tag{8}$$

Introduction of λ ensures that the matrix $(\boldsymbol{R}_{pr(i)}\boldsymbol{R}_{pr(i)}^T + \lambda I)$ is always positive definite regardless of the rank of $\boldsymbol{R}_{pr(i)}$. This allows one to draw inference on different variables of Eq. 1 in a Bayesian setting even when $n_p^i < n_k^i$. Choosing an appropriate value of the proportionality constant c is the topic of much debate [1]. The most comprehensive way of choosing c is to estimate its posterior distribution based on experimental data [1]. However, in this paper we resort to a simple but intuitive choice of $c = n_p^i$ drawing on the notion that the "amount of information" contained in the prior equalize the amount of information in one observation. The value of λ was arbitrarily chosen to be 0.1 since it was found that any reasonable value within the range $0 < \lambda < 1$ works well[2].

The connection coefficients which do not represent true interactions, i.e. $r_{ij} \notin \boldsymbol{\rho}_i$ are assumed to be degenerate random variables which are 0 with probability 1. The resulting prior distribution of the connection coefficients $\boldsymbol{r}_i$ is called spike and slab prior. The distribution of $\boldsymbol{\rho}_i$ forms the slab and the distributions of $r_{ij} \in \boldsymbol{\rho}_i$ form the spikes. In summery, the prior distribution of $\boldsymbol{r}_i$, denoted by $P(\boldsymbol{r}_i|\boldsymbol{A}_i, \sigma^2)$ is given below:

$$\begin{aligned} \boldsymbol{\rho}_i &\sim N(\mathbf{0}, c\sigma^2(\boldsymbol{R}_{pr(i)}\boldsymbol{R}_{pr(i)}^T + \lambda I)^{-1}) \\ P(r_{ij} = 0) &= 1, \text{if } r_{ij} \notin \boldsymbol{\rho}_i \end{aligned} \tag{9}$$

Finally the noise ϵ_{ik} is assumed to be a zero mean normal(Gaussian) variable with variance σ^2. Here, the noise variance represents our prior assumption about the extent of uncertainty present in the data. The extent of uncertainty in a biological datasets depends both on the biological system being analyzed and the measurement system used in the experiments. Therefore, we assumed that σ itself is a stochastic parameter which has an inverse gamma distribution with parameters α and β, i.e.

$$\sigma^2|\alpha,\beta \sim IG(\alpha,\beta) \tag{10}$$

Here, we have assumed $\alpha = 1$ and $\beta = 1$ to ensure that σ^2 has a flat distribution implying that the extent of noise may vary significantly depending on biological and experimental factors. We shall refer to this distribution as $P(\sigma^2)$ hereafter for notational conveniences. The relationship between the observed data, the unknown variables, the parameters and hyper parameters of our Bayesian framework is shown in a graphical model in Fig. **??**.

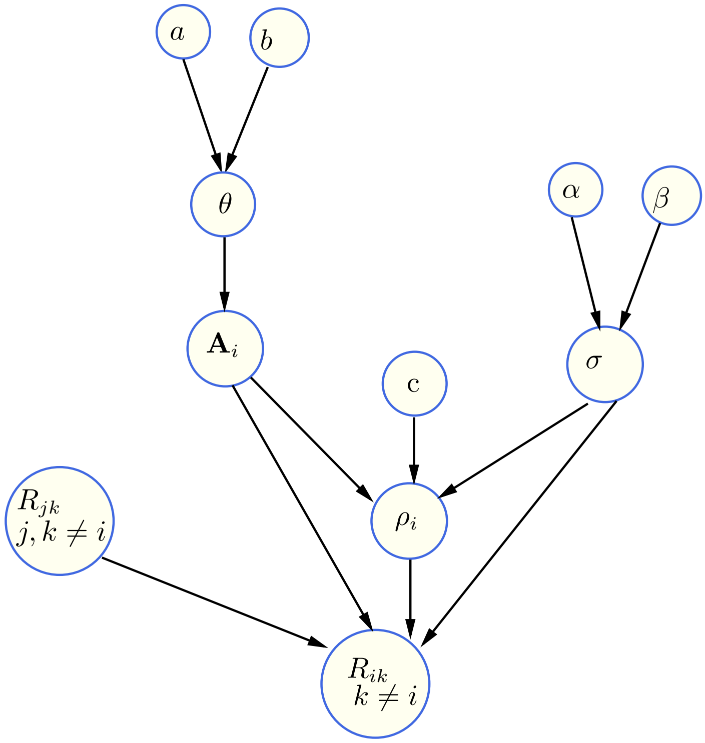

The likelihood function and the marginal likelihood: The likelihood function is the probability distribution of the global responses($\boldsymbol{R}_i$) of node i given the global responses of its potential regulators ($\boldsymbol{R}_{pr(i)}$), the connection coefficients $\boldsymbol{\rho}_i$ and the noise variance σ^2. The likelihood of the model shown in Eq.2 is given by:

$$P(\boldsymbol{R}_i|\boldsymbol{R}_{pr(i)}, \boldsymbol{r}_i, \boldsymbol{A}_i, \sigma^2) = N(\boldsymbol{R}_{pr(i)}^T \boldsymbol{\rho}_i, \sigma^2 \boldsymbol{I}) \tag{11}$$

In this paper, we are interested in inferring the potential regulators of a node i, i.e. $\boldsymbol{A}_i$. Therefore, we calculated the marginal likelihood of $\boldsymbol{A}_i$ marginalizing the likelihood function with respect to $\boldsymbol{\rho}_i$ and σ^2 as shown below:

$$\begin{aligned}
P(\boldsymbol{R}_i|\boldsymbol{R}_{pr(i)}, \boldsymbol{A}_i) &= \oint_{\boldsymbol{r}_i} \int_{\sigma^2} P(\boldsymbol{R}_i|\boldsymbol{R}_{pr(i)}, \boldsymbol{r}_i, \boldsymbol{A}_i, \sigma^2) P(\boldsymbol{r}_i|\boldsymbol{A}_i, \sigma^2) P(\sigma^2) d\boldsymbol{r}_i d\sigma^2 \qquad (12) \\
&= \oint_{\boldsymbol{r}_i} \int_{\sigma^2} N(\boldsymbol{R}_{pr(i)}^T \boldsymbol{\rho}_i, \sigma^2 \boldsymbol{I}) N(\boldsymbol{0}, c\sigma^2 (\boldsymbol{R}_{pr(i)} \boldsymbol{R}_{pr(i)}^T + \lambda I)^{-1} IG(\alpha, \beta) d\boldsymbol{r}_i d\sigma^2 \\
&= \frac{1}{\sqrt{(2\pi)}^{\frac{n_p^i}{2}}} c^{\frac{-n_k^i}{2}} \frac{\beta^\alpha}{\Gamma(\alpha)} \Gamma(\alpha + \frac{n_p^i}{2}) \frac{|\boldsymbol{R}_{pr(i)} \boldsymbol{R}_{pr(i)}^T|^{\frac{1}{2}}}{|\frac{(\boldsymbol{R}_{pr(i)} \boldsymbol{R}_{pr(i)}^T + \lambda I)}{c} + \boldsymbol{R}_{pr(i)} \boldsymbol{R}_{pr(i)}^T|^{\frac{1}{2}}} \times \\
&\quad \left(\beta + \frac{1}{2} \left(\boldsymbol{R}_i {\boldsymbol{R}_i}^T - \boldsymbol{R}_i {\boldsymbol{R}_{pr(i)}}^T \left(\frac{(\boldsymbol{R}_{pr(i)} \boldsymbol{R}_{pr(i)}^T + \lambda I)}{c} + \boldsymbol{R}_{pr(i)} \boldsymbol{R}_{pr(i)}^T \right)^{-1} \boldsymbol{R}_{pr(i)} {\boldsymbol{R}_i}^T \right) \right)^{-(\frac{n_p^i}{2} + \alpha)}
\end{aligned}$$

The posterior distribution: The marginal posterior of $\boldsymbol{A}_i$ was calculated from the marginal likelihood using Bayes' theorem as shown below.

$$\begin{aligned}
P(\boldsymbol{A}_i|\boldsymbol{R}) &= \frac{P(\boldsymbol{R}_i|\boldsymbol{R}_{pr(i)}, \boldsymbol{A}_i) P(\boldsymbol{A}_i)}{P(\boldsymbol{R}_i|\boldsymbol{R}_{pr(i)})} \qquad (13) \\
&= \frac{P(\boldsymbol{R}_i|\boldsymbol{R}_{pr(i)}, \boldsymbol{A}_i) P(\boldsymbol{A}_i)}{\sum_{\boldsymbol{A}_i} P(\boldsymbol{R}_i|\boldsymbol{R}_{pr(i)}, \boldsymbol{A}_i) P(\boldsymbol{A}_i)}
\end{aligned}$$

The denominator in Eq.14 has to be calculated numerically which is possible for small networks (typically $n \leq 20$) using computers. For large networks, this becomes prohibitively computation intensive. In case of large networks, one can calculate $P(\boldsymbol{A}_i|\boldsymbol{R})$ up to a constant of proportionality, i.e.

$$
\begin{aligned}
P(\boldsymbol{A}_i|\boldsymbol{R}) &\propto P(\boldsymbol{R}_i|\boldsymbol{R}_{pr(i)},\boldsymbol{A}_i)P(\boldsymbol{A}_i) \\
&\propto c^{\frac{-n_k^i}{2}} \frac{|\boldsymbol{R}_{pr(i)}\boldsymbol{R}_{pr(i)}^T|^{\frac{1}{2}}}{|\frac{(\boldsymbol{R}_{pr(i)}\boldsymbol{R}_{pr(i)}^T+\lambda I)}{c}+\boldsymbol{R}_{pr(i)}\boldsymbol{R}_{pr(i)}^T|^{\frac{1}{2}}} \times \\
&\quad \left(\beta+\frac{1}{2}\left(\boldsymbol{R}_i{\boldsymbol{R}_i}^T-\boldsymbol{R}_i{\boldsymbol{R}_{pr(i)}}^T\left(\frac{(\boldsymbol{R}_{pr(i)}\boldsymbol{R}_{pr(i)}^T+\lambda I)}{c}+\boldsymbol{R}_{pr(i)}\boldsymbol{R}_{pr(i)}^T\right)^{-1}\boldsymbol{R}_{pr(i)}{\boldsymbol{R}_i}^T\right)\right)^{-(\frac{n_p^i}{2}+\alpha)} \times \\
&\quad \binom{(n-1)}{n_k^i} Beta(a+n_k^i, b+n-1-n_k^i)
\end{aligned} \tag{14}
$$

Inferences can be drawn about different properties of $\boldsymbol{A}_i$ by sampling from the marginal posterior shown above.

References

[1] M. Feldkircher and S. Zeugner. Benchmark priors revisited: On adaptive shrinkage and the supermodel effect in bayesian model averaging. Technical report, IMF, 2009.

[2] M. Gupta and J.G. Ibrahim. An information matrix prior for bayesian analysis in generalized linear models with high dimensional data. *Stat Sin*, 19:16411663., 2009.

[3] B. Kholodenko, A. Kiyatkin, F. J. Bruggeman, E. Sontag, and J. B. Westerhoff, H. V. Hoek. Untangling the wires: A strategy to trace functional interactions in signaling and gene networks. *PNAS*, 99:12841–12846, 2002.

[4] A. Zellner. *On assessing prior distributions and Bayesian regression analysis with g-prior distributions, volume Bayesian Inference and Decision Techniques: Essays in Honor of Bruno de Finetti. North-Holland, Amsterdam.* 1986.
