# Supplementary material for: Integrating Bayesian variable selection with Modular Response Analysis to infer biochemical network topology
Source: BMC Syst Biol. 2013 Jul 6;7:57. doi: 10.1186/1752-0509-7-57 (PMC3726398; doi:10.1186/1752-0509-7-57)
Supplement: Additional file 3 — MAPK model. In this file, we have provided the details of the ODE and the SDE models which were created to simulate the noise free and noisy perturbation response of the MAPK pathway respectively. [file 1752-0509-7-57-S3.pdf]

The MAPK model

Tapesh Santra, Walter Kolch, Boris N. Kholodenko

...

ODE simulation: The ODE model for our simulation experiment is shown in Tab.1.

Table 1: MAPK model

**Receptor module**		
EGF binds to EGFR	R + L = RL	$ka = 0.001, kd = 0.01$
EGF-EGFR complex dimerization	RL + RL = RD	$ka = 0.001, kd = 0.01$
EGF-EGFR complex phosphorylation	RD→ pRD	$k = 0.01$
EGFR complex dephosphorylation	pRD→ RD	$\frac{Vm_1}{(Km_1+pRD)}$; $Vm_1 = 1, Km_1 = 10$
**Adapter module**		
Phosphorylation of Shc by EGFR	Shc→ pShc; pRD	$\frac{kcat_1 \times pRD \times Shc}{(K_1+Shc)}$; $kcat_1 = 0.1, K_1 = 5$
Dephosphorylation of Shc	pShc→ Shc	$\frac{Vm_2 \times pShc}{(Km_2+pShc)}$; $Vm_2 = 0.08, Km_2 = 200$
Shc binds to Grb2SOS complex	pShc + GS = pShc-GS	$ka = 0.001, kd = 0.01$
ppERK phosphorylates and inhibits GS	GS→ iGS; ppERK	$\frac{kcat_9 \times GS \times ppERK}{(K_9+GS)}$; $kcat_9 = 0.01, K_9 = 10$
iGS dephosphorylation	iGS→ GS	$\frac{Vm_{10} iGS}{(Km_{71}+iGS)}$; $Vm_{10} = 0.05, Km_{71} = 10$
**Initiator module**		
Phosphorylation of RasGDP by Shc bound Grb2SOS	RasGDP→ RasGTP; pShc-GS	$\frac{kcat_2 \times pShc \times GS \times RasGDP}{(K_2+RasGDP)}$; $kcat_2 = 0.001, K_2 = 10$
Dephosphorylation of RasGTP	RasGTP→ RasGDP	$\frac{Vm_3 RasGTP}{(Km3+RasGTP)}$; $Vm_3 = 0.1, Km_3 = 5$
**MAP3K module**		
Raf activation stage 1	Raf→ aRaf; RasGTP, ppERK	$\frac{kcat_3 \times RasGTP \times Raf}{((K_{31}+Raf+aRaf\frac{K_{31}}{K_{32}})(1+\frac{ppERK}{K_i}))}$; $kcat_3 = 0.1, K_{31} = 300, K_{32} = 20; K_i = 5$
Raf activation stage 2	aRaf→ aaRaf; RasGTP, ppERK	$\frac{kcat_4 \times RasGTP \times aRaf}{((K_{31}+Raf+aRaf\frac{K_{31}}{K_{32}})(1+\frac{ppERK}{K_i}))}$; $kcat_4 = 0.1, K_{31} = 300, K_{32} = 20; K_i = 5$
Raf deactivation stage 1	aaRaf→ aRaf;	$\frac{Vm_4 \times aaRaf}{(Km_{41}+aaRaf+(aRaf\frac{Km_{41}}{Km_{42}})+Raf\frac{Km_{41}}{Km_{43}})}$; $Vm_4 = 0.05, Km_{41} = 22, Km_{42} = 18; Km_{43} = 80$
Raf deactivation stage 1	aRaf→ Raf;	$\frac{Vm_5 \times aRaf}{(Km_{41}+aaRaf+(aRaf\frac{Km_{41}}{Km_{42}})+Raf\frac{Km_{41}}{Km_{43}})}$; $Vm_5 = 0.05, Km_{41} = 22, Km_{42} = 18; Km_{43} = 80$
**MAP2K module**		
MEK phosphorylation	MEK→pMEK; aaRaf	$\frac{kcat_5 \times MEK \times aaRaf}{(K_{41}+MEK+pMEK\frac{K_{41}}{/}K_{42})}$; $kcat_5 = 0.1, K_{41} = 300; K_{42} = 20$
MEK doublephosphorylation	pMEK → ppMEK; aaRaf	$\frac{kcat_6 \times pMEK \times aaRaf}{(K41+MEK+pMEK\frac{K41}{K42})}$; $kcat_6 = 0.1, K_{41} = 300; K_{42} = 20$
MEK dephosphorylation stage 1	ppMEK→ pMEK; ppERK	$\frac{Vm_6 \times ppMEK \times (1+A\frac{ppERK}{Kmp})}{((Km_{51}+ppMEK+(pMEK\frac{Km_{51}}{Km_{52}})+MEK\frac{Km_{51}}{Km_{53}})*(1+ppERK/Kmp))}$; $Vm_6 = 0.09, Km_{51} = 22, Km_{52} = 18; Km_{53} = 80; Kmp = 100$
MEK dephosphorylation stage 2	pMEK→ MEK; ppERK	$\frac{Vm_7 \times pMEK \times (1+A\frac{ppERK}{Kmp})}{((Km_{51}+ppMEK+pMEK\frac{Km_{51}}{Km_{52}}+MEK\frac{Km_{51}}{Km_{53}})(1+\frac{ppERK}{Kmp}))}$; $Vm_7 = 0.09, Km_{51} = 22, Km_{52} = 18; Km_{53} = 80; Kmp = 100$
**MAPK module**		
ERK phosphorylation	ERK→ pERK; ppMEK	$\frac{kcat_7 \times ERK \times ppMEK}{(K_{51}+ERK+pERK\frac{K_{51}}{K_{52})}}$; $kcat_7 = 0.1, K_{51} = 300; K_{52} = 20$
ERK doublephosphorylation	pERK→ ppERK; ppMEK	$\frac{kcat_8 \times pERK \times ppMEK}{(K_{51}+ERK+pERK\frac{K_{51}}{K_{52})}}$; $kcat_8 = 0.1, K_{51} = 300; K_{52} = 20$
ERK dephosphorylation stage 1	ppERK→ pERK	$\frac{Vm_8 \times ppERK}{(Km_{61}+ppERK+pERK\frac{Km_{61}}{Km_{62}}+ERK\frac{Km_{61}}{Km_{63}})}$; $Vm_8 = 0.05, Km_{61} = 22, Km_{62} = 18, Km_{63} = 80$
ERK dephosphorylation stage 2	pERK→ ERK	$\frac{Vm_9 \times pERK}{(Km_{61}+ppERK+pERK\frac{Km_{61}}{Km_{62}}+ERK\frac{Km_{61}}{Km_{63})})}$; $Vm_9 = 0.05, Km_{61} = 22, Km_{62} = 18, Km_{63} = 80$

The initial concentrations for the MAPK model are given in Tab.2.

siRNA knockdown experiments siRNA knockdown of a gene results in attenuated level of the corresponding proteins. Therefore we simulated knockdown experiments by reducing the initial concentrations of the corresponding proteins. The initial concentrations regarding the knock down experiments are shown in Tab.3.

Table 2: Initial concentrations for the MAPK model

Intial concentrations
L=1
R=100
SHC = 300
GS=100
RasGDP =100
RAF_total= Raf + aRaf+ aaRaf= 100
MEK_total = MEK + pMEK + ppMEK=100
ERK_total = ERK + pERK + ppERK=100

Table 3: Initial concentrations for the knockdown experiments

**Replicate 1: 80% knockdown efficientcy**						
Module:	Exp. no.					
	1	2	3	4	5	6
R	20	100	100	100	100	20
Shc	300	60	300	300	300	300
RasGDP	100	100	20	100	100	100
RAFtotal	100	100	100	20	100	100
MEKtotal	100	100	100	100	20	100
ERKtotal	100	100	100	100	100	20
**Replicate 2: 60% knockdown efficiency**						
Module:	Exp. no.					
	1	2	3	4	5	6
R	40	100	100	100	100	20
Shc	300	120	300	300	300	300
RasGDP	100	100	40	100	100	100
RAFtotal	100	100	100	40	100	100
MEKtotal	100	100	100	100	40	100
ERKtotal	100	100	100	100	100	40
**Replicate 3: 40% knockdown efficiency**						
Module:	Exp. no.					
	1	2	3	4	5	6
R	60	100	100	100	100	20
Shc	300	180	300	300	300	300
RasGDP	100	100	60	100	100	100
RAFtotal	100	100	100	60	100	100
MEKtotal	100	100	100	100	60	100
ERKtotal	100	100	100	100	100	60

SDE simulation: Each ordinary differential equation of the following form

$$\frac{dx}{dt} = V(\boldsymbol{x}) - D(\boldsymbol{x}) \tag{1}$$

was converted into the a stochastic differential equation of the following form [1]

$$\frac{dx}{dt} = V(\boldsymbol{x}) - D(\boldsymbol{x}) + c_1 \left(\sqrt{V(\boldsymbol{x})}\eta_v + \sqrt{D(\boldsymbol{x})}\eta_d \right) \tag{2}$$

Here, η_v and η_d are independent Gaussian white noises and $c_1 = 0.01$ is a multiplicative constant which controls the amplitude of the molecular noise. The scheme was adopted from [1].

References

[1] T. Schaffter, D. Marbach, and Floreano D. Genenetweaver: In silico benchmark generation and performance profiling of network inference methods. *Bioinformatics*, 26:2263–70, 2011.
