# Supplementary material for: Integrating Bayesian variable selection with Modular Response Analysis to infer biochemical network topology
Source: BMC Syst Biol. 2013 Jul 6;7:57. doi: 10.1186/1752-0509-7-57 (PMC3726398; doi:10.1186/1752-0509-7-57)
Supplement: Additional file 4 — Figure S1. In this figure, we have illustrated the convergence of the Gibbs samplers which were created to reconstruct the MAPK pathway from noise free simulation data. [file 1752-0509-7-57-S4.pdf]

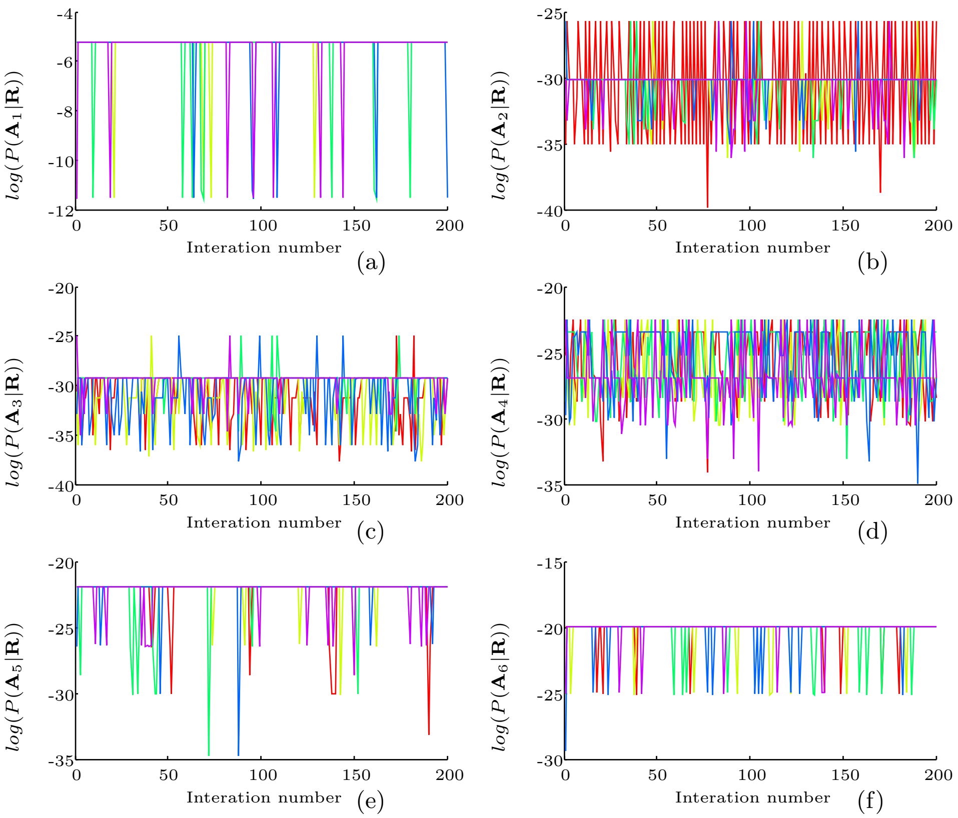

Figure S1: Convergence of the Gibbs samplers for noisy free data. The log-marginals of the samples are plotted in the Y-axis and the corresponding iteration numbers is plotted in X-axis. (a) shows the log-marginals of samples produced by 5 parallel samplers (indicated by five different colors) searching for the potential regulators of the receptor (EGFR) module. (b),(c),(d),(e)and (f) show the same for the adaptor (Grb2SOS), initiator(Ras), MAP3K(RAF), MAP2K(MEK) and MAPK(ERK) modules respectively. It can be noticed that in each subplot, the lines of different colors cover the same area in the plot, suggesting that the parallel samplers were sampling from the same distribution. If the samplers were sampling from different distribution one would expect the lines of different colors not to overlap on each other.
