# Supplementary material for: Integrating Bayesian variable selection with Modular Response Analysis to infer biochemical network topology
Source: BMC Syst Biol. 2013 Jul 6;7:57. doi: 10.1186/1752-0509-7-57 (PMC3726398; doi:10.1186/1752-0509-7-57)
Supplement: Additional file 8 — References for ERBB pathway. In this file we have provided the references for different interactions of the ERBB pathway. [file 1752-0509-7-57-S8.pdf]

Supplementary material: The ERBB pathway that regulates the G1/S transition

Tapesh Santra, Walter Kolch, Boris N. Kholodenko

October 23, 2012

Edge (1): ERBB weakly activates PI3K kinase which phosphorylates AKT [3]

Edge (2): ERBB phosphorylates ERK by activating RAF/MEK/ERK pathway [15].

Edge (3): There are several possible mechanisms by which ERK pathway may regulate the activity of ERBB receptors. One such mechanism involves the Eearly Growth response genes such as EGR-1. In response to EGFR, ERK pathway activates the transcription of EGR-1. EGR1 can directly bind to the promoter of TGF-α [25] which activates ERBB, thus forming a positive feedback loop. These feedback loops are known as autocrine loops and implicated for drug resistance in many different cancer cells [24]. In another mechanism, EGR-1 binds directly to the promoter of the EGFR gene and inhibits its transcription, thus forming a negative feedback loop[1] (not shown in the paper).

Edge (4): AKT, a downstream kinase of ERBB, inhibits ADAM17 which activates TGF-α[12].

Edge (5): Regulation of Retinoblastoma protein by ERBB in an AKT and ERK independent manner, possibly via JNK pathway [10].

Edge (6): p27 can be regulated by ERBB in an ERK or AKT independent manner, possibly via Src [7].

Edge (7): CDK2/Cyclin-A complex phosphorylates and activates CDC25 [20] which is a phosphatase of ERK [28].

Edge (8): ERK phosphorylates and inhibits the activity of IRS [11] which is a key adapter protein for ERBB mediated AKT activation [3] .

Edge (9): AKT is found to stabilize Cyclin D1, since inhibiting PI3K decreases the half-life of Cyclin D1 [8].

Edge (10): ERK pathway transcriptionally activates Cyclin-D1 [29]

Edge (11): ERK pathway regulates C-MYC expression [17] and C-MYC functionally antagonizes the action of p27 [18].

Edge (12): ERK pathway regulates the activity of CDK2 in many different ways. ERK positively regulates the expression of Cyclin D and E which binds with CDK2 and induces its kinase activity [16]. ERK also induces the nuclear localization of CDK2 and phosphorylates it at the activating site Thr160 [16].

Edge (13): PI3K pathway regulates the protein stability of C-MYC [26] and C-MYC functionally antagonizes the action of p27 [18].

Edge (14): PI3K pathway regulates the protein stability of C-MYC [26] and C-MYC inhibits p21 [22].

Edge (15): p21 binds to the promoter region of CDC25A and inhibits its transcription [27]. CDC25A acts as a phosphatase and dephosphorylates ERK [28].

Edge (16): ERK pathway regulates C-MYC expression [17] and C-MYC inhibits p21 [22].

Edge (17): p21 activates PDK1 which mediates the expression of Cyclin D1 [2].

Edge (18): CDK4 regulates the nuclear translocation and kinase activity of CDK2 [30].

Edge (19): p27 inhibits the activity of CDK2 [5].

Edge (20): p27 inhibits the activity of CDK4 [5].

Edge (21): p21 inhibits the activity of CDK4 [14].

Edge (22): p21 inhibits the activity of CDK2 [14].

Edge (23): The growth factor activation of Cyclin D/CDK4 complex sequesters unbound p21 and inhibits its inhibitory effect of Cyclin E/CDK2 complex.

Edge (24): The growth factor activation of Cyclin D/CDK4 complex sequesters unbound p27 and inhibits its inhibitory effect of Cyclin E/CDK2 complex.

Edge (25): Cyclin D1 binds to CDK4 to activate its kinase activity [21].

Edge (26): Cyclin E/CDK2 complex phosphorylates p27 and promotes its degradation.

Edge (27): CDK2/Cyclin-E complex phosphorylates pRB1 [13].

Edge (28): CDK4/Cyclin-D complex phosphorylates pRB1 [13].

Edge (29): pRB phosphorylation activates E2F transcription factors which activate ARF. ARF regulates p53 protein [23]. p53 inhibits the translation of the CDK4 protein [9] forming a feedback loop.

Edge (30): pRB phosphorylation activates E2F transcription factors which directly binds to the promoters of CDK2 [4].

Edge (31): Cyclin D1 binds to CDK6 to form a heterodimer. The heterodimer complex phosphorylates pRB.

Edge (32): pRB phosphorylation activates E2F transcription factors which binds to AKT1 promoter and activates its transcription [6].

Edge (33): CDK2 regulates apoptin which modulates the activity of AKT by regulating its subcellular localization.

Edge (34): pRB phosphorylation activates E2F transcription factors, E2F transcriptionally activates PAC1 which dephosphorylates ERK [31] completing a negative feedback loop.

Edge (35): ERK phosphorylates and activates MDM2 [19] which binds with pRB and modulates its activity [23].

References

[1] S. Arora, Y. Wang, Z. Jia, A. Vardar-Sengul, A. Munawar, K. S. D. Doctor, M. Birrer, M. McClelland, E. Adamson, and D. Mercola. Egr1 regulates the coordinated expression of numerous egf receptor target genes as identified by chip-on-chip. *Genome Biology*, 9:R166, 2008.

[2] S. Balasenthil, A.A. Sahin, C. J. Barnes, R. A. Wang, R.G. Pestell, R.K. Vadlamudi, and R. Kumar. p21-activated kinase-1 signaling mediates cyclin d1 expression in mammary epithelial and cancer cells. *J Biol Chem*, 279:1422–1428, 2004.

[3] N. Borisov, E. Aksamitiene, A. Kiyatkin, S. Legewie, J. Berkhout, T. Maiwald, N. P. Kaimachnikov, J. Timmer, J.B. Hoek, and B.N. Kholodenko. Systems-level interactions between insulinegf networks amplify mitogenic signaling. *Molecular Systems Biology*, 5:256, 2009.

[4] A. P. Bracken, M. Ciro, A. Cocito, and C. Helin. E2f target genes: unraveling the biology. *Trends in Biochemical Sciences*, 29:409–417, 2004.

[5] S. Cariou, J.C.H. Donovan, W. M. Flanagan, A. Milic, N. Bhattacharya, and J.M. Slingerland. Down-regulation of p21waf1ycip1 or p27kip1 abrogates antiestrogen-mediated cell cycle arrest in human breast cancer cells. *PNAS*, 97:90429046, 2000.

[6] M. Chaussepied and D. Ginsberg. Transcriptional regulation of akt activation by e2f. *Molecular Cell*, 16:831–837, 2004.

[7] I. Chu, J. Sun, A. Arnaout, H. Kahn, W. Hanna, S. Narod, P. Sun, C. Tan, L. Hengst, and J. Slingerland. p27 phosphorylation by src regulates inhibition of cyclin e-cdk2 and p27 proteolysis. *Cell*, 128:281294., 2007.

[8] B. Dufourny, H. A. A. M. Van Teeffelen, I.H.L. Hamelers, J.S. Sussenbach, and P. H. Steenbergh. Stabilization of cyclin d1 mrna via the phosphatidylinositol 3-kinase pathway in mcf-7 human breast cancer cells. *Journal of Endocrinology*, 166:329338, 2000.

[9] M.E. Ewen, C.J. Oliver, H. K. Sluss, S. J. Miller, and D. S. Peeper. p53-dependent repression of cdk4 translation in tgf-beta-induced g1 cell-cycle arrest. *Genes Dev*, 9:204–217, 1995.

[10] D. Faust, I. Dolado, A. Cuadrado, F. Oesch, C. Weiss, A. R. Nebreda, and C. Dietrich. p38alpha mapk is required for contact inhibition. *Oncogene*, 24:7941–7945, 2005.

[11] L. Fritsche, S. S. Neukamm, R. Lehmann, E. Kremmer, A. M. Hennige, A.H. Gugel, M. Schenk, E.D. Haring, H. U.and Schleicher, and C. Weigert. Insulin-induced serine phosphorylation of irs-2 via erk1/2 and mtor: studies on the function of ser675 and ser907. *Am J Physiol Endocrinol Metab*, 300:E824E836, 2011.

[12] M. Gijsen, P. King, T. Perera, P. J. Parker, A. L. Harris, B. Larijani, and A. Kong. Her2 phosphorylation is maintained by a pkb negative feedback loop in response to anti-her2 herceptin in breast cancer. *PLoS Biology*, 8:e1000563, 2010.

[13] J. W. Harbour and D. C. Dean. The rb/e2f pathway: expanding roles and emerging paradigms. *Gene*, 14:2393–2409, 2000.

[14] J.W. Harper et al. Inhibition of cyclin-dependent kinases by p21. *Mol Biol Cell*, 6:387–400., 1995.

[15] W. Kolch. Meaningful relationships : the regulation of the ras/raf/mek/erk pathway by protein interactions. *Biochem. J.*, 351:289–305, 2000.

[16] N.H. Lents, S.M. Keenan, C. Bellone, and J.J. Baldassare. Stimulation of the raf/mek/erk cascade is necessary and sufficient for activation and thr-160 phosphorylation of a nuclear-targeted cdk2. *J Biol Chem*, 277:49, 2002.

[17] M. M. Liu, C. Albanese, C. M. Anderson, K. Hilty, P. Webb, R. M. Uht, R. H. Price, R. G. Pestell, and P. J. Kushner. Opposing action of estrogen receptors alpha and beta on cyclin d1 gene expression. *J. Biol Chem*, 277:24353–24360, 2002.

[18] S. Maddika, S.R. Ande, S. Panigrahi, et al. Cell survival, cell death and cell cycle pathways are interconnected: Implications for cancer therapy. *Drug Resist. Update*, 10:13–29, 2007.

[19] M. Malmlof, E. Roudier, J. Hgberg, and U. Stenius. Mek-erk-mediated phosphorylation of mdm2 at ser-166 in hepatocytes. mdm2 is activated in response to inhibited akt signaling. *J Biol Chem*, 282:2288–2296, 2007.

[20] J. Mitra and G. H. Enders. Cyclin a/cdk2 complexes regulate activation of cdk1 and cdc25 phosphatases in human cells. *Oncogene*, 23:33613367, 2004.

[21] D. O. Morgan. Cyclin-dependent kinases: engines, clocks, and microprocessors. *Annu Rev Cell Dev Biol*, 13:261–91., 1997.

[22] S. Pelengaris, M. Khan, and G. Evan. c-myc: more than just a matter of life and death. *Nature Reviews Cancer*, 2:764–776, 2002.

[23] S. Polager and D. Ginsberg. p53 and e2f: partners in life and death. *Nature Reviews*, 9:738–748, 2009.

[24] C. A. Ritter, R. M. Perez-Torres, C. Rinehart, M. Guix, T. Dugger, J. A. Engelman, and C. L. Arteaga. Human breast cancer cells selected for resistance to trastuzumab in vivo overexpress epidermal growth factor receptor and erbb ligands and remain dependent on the erbb receptor network. *Clinical Cancer Reasearch*, 13:4909, 2007.

[25] L. Sauer, D. Gitenay, C. Vo, and V. T. Baron. Mutant p53 initiates a feedback loop that involves egr-1/egf receptor/erk in prostate cancer cells. *Oncogene*, 29:2628, 2010.

[26] R. Sears, F. Nuckolls, E. Haura, Y. Taya, Y. Tamai, and J. Nevins. Multiple ras-dependent phosphorylation pathways regulate myc protein stability. *Genes and Dev.*, 14:25012514, 2000.

[27] A. Vigneron, J. Cherier, B. Barre, E. Gamelin, and O. Coqueret. The cell cycle inhibitor p21waf1 binds to the myc and cdc25a promoters upon dna damage and induces transcriptional repression*. *J. Biol Chem*, 281:3474234750,, 2006.

[28] Z Wang, B. Zhang, M. Wang, and B. I. Carr. Cdc25a and erk interaction: Egfr-independent erk activation by a protein phosphatase cdc25a inhibitor, compound 5. *J Cell Physiol*, 204:437–444, 2005.

[29] J. D. Weber, D. M. Raben, P. J. Phillips, and J. J. Baldassare. Sustained activation of extracellular-signal-regulated kinase 1 (erk1) is required for the continued expression of cyclin d1 in g1 phase. *Biochem. J.*, 326:61–68., 1997.

[30] L. Wierod, C. M. Rosseland, B. Lindeman, M.P. Oksvold, H. Grosvik, E. Skarpen, and H. S. Huitfeldt. Cdk2 regulation through pi3k and cdk4 is necessary for cell cycle progression of primary rat hepatocytes. *Cell Proliferation*, 40:475487, 2007.

[31] J. Wu, Y. J. Jin, G. M. Calaf, W. L. Huang, and Y. Yin. Pac1 is a direct transcription target of e2f-1 in apoptotic signaling. *Oncogene*, 26:6526–6535, 2007.
