# Supplementary material for: Integrating Bayesian variable selection with Modular Response Analysis to infer biochemical network topology
Source: BMC Syst Biol. 2013 Jul 6;7:57. doi: 10.1186/1752-0509-7-57 (PMC3726398; doi:10.1186/1752-0509-7-57)
Supplement: Additional file 9 — Figure S3. In this figure, we have illustrated the convergence of the Gibbs samplers which were created to reconstruct the ERBB-G1/S transition pathway from experimentally obtained perturbation data. [file 1752-0509-7-57-S9.pdf]

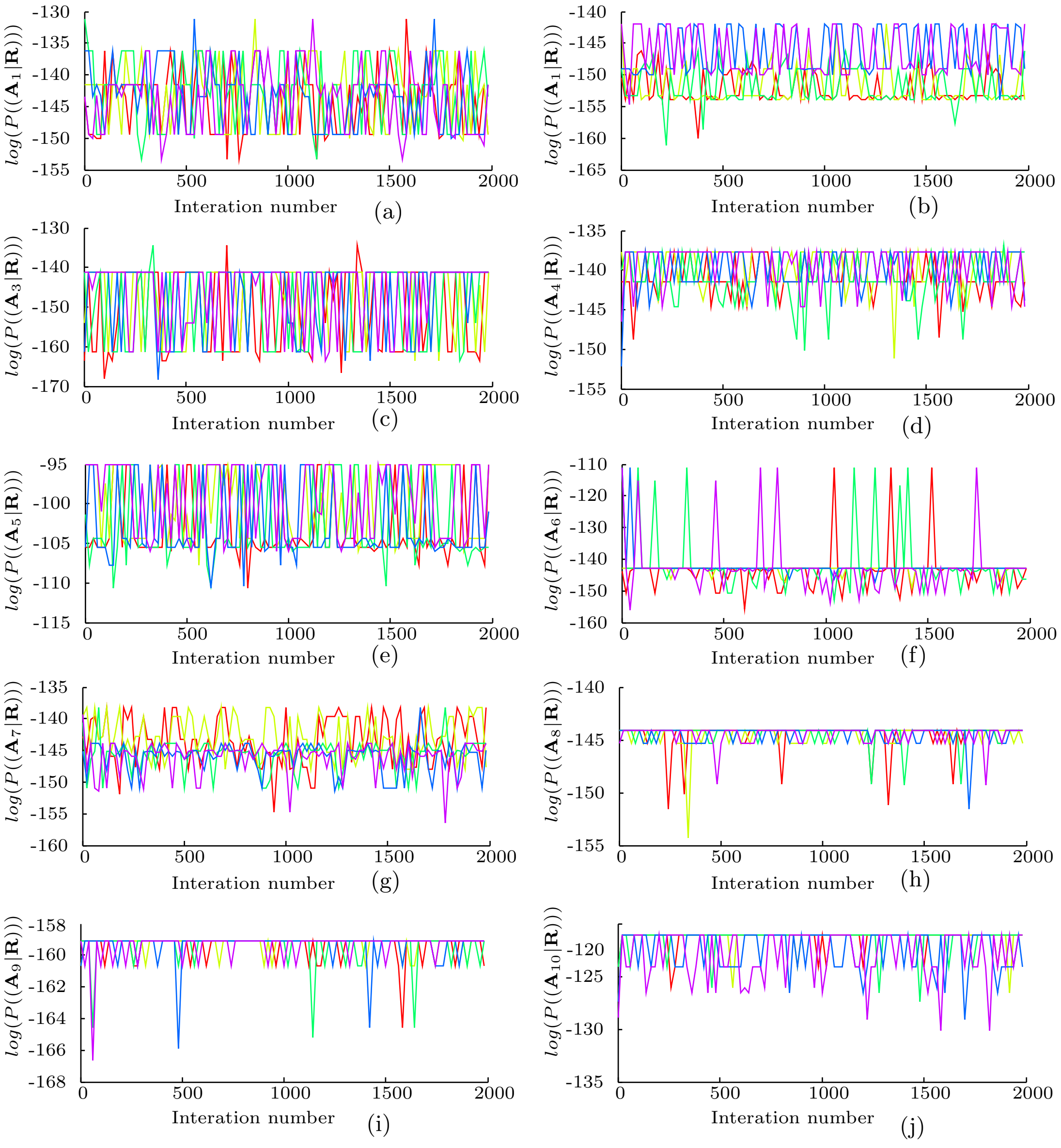

Figure S3: Convergence of the Gibbs samplers for perturbation data obtained by perturbing the ERBB regulated G1/S transition pathway. The log-marginals of the samples are plotted in the Y-axis and the corresponding iteration numbers are plotted in X-axis. (a) shows the log-marginals of samples produced by 5 parallel samplers (indicated by five different colors) searching for the potential regulators of the ERBB1. (b)-(j) show the same for ERBB2, p21,p27, Cyclin-D1, CDK2, CDK4,AKT, ERK and pRB1. It can be noticed that in each subplot, the lines of different colors cover the same area in the plot, suggesting that the parallel samplers were sampling from the same distribution. If the samplers were sampling from different distributions one would expect the lines of different colors not to overlap on each other.
