# Supplementary material for: Integrating Bayesian variable selection with Modular Response Analysis to infer biochemical network topology
Source: BMC Syst Biol. 2013 Jul 6;7:57. doi: 10.1186/1752-0509-7-57 (PMC3726398; doi:10.1186/1752-0509-7-57)
Supplement: Additional file 10 — Figure S4. In this figure, we have shown the topology of the ERBB-G1/S transition network as reconstructed by the Median Probability Model. [file 1752-0509-7-57-S10.pdf]

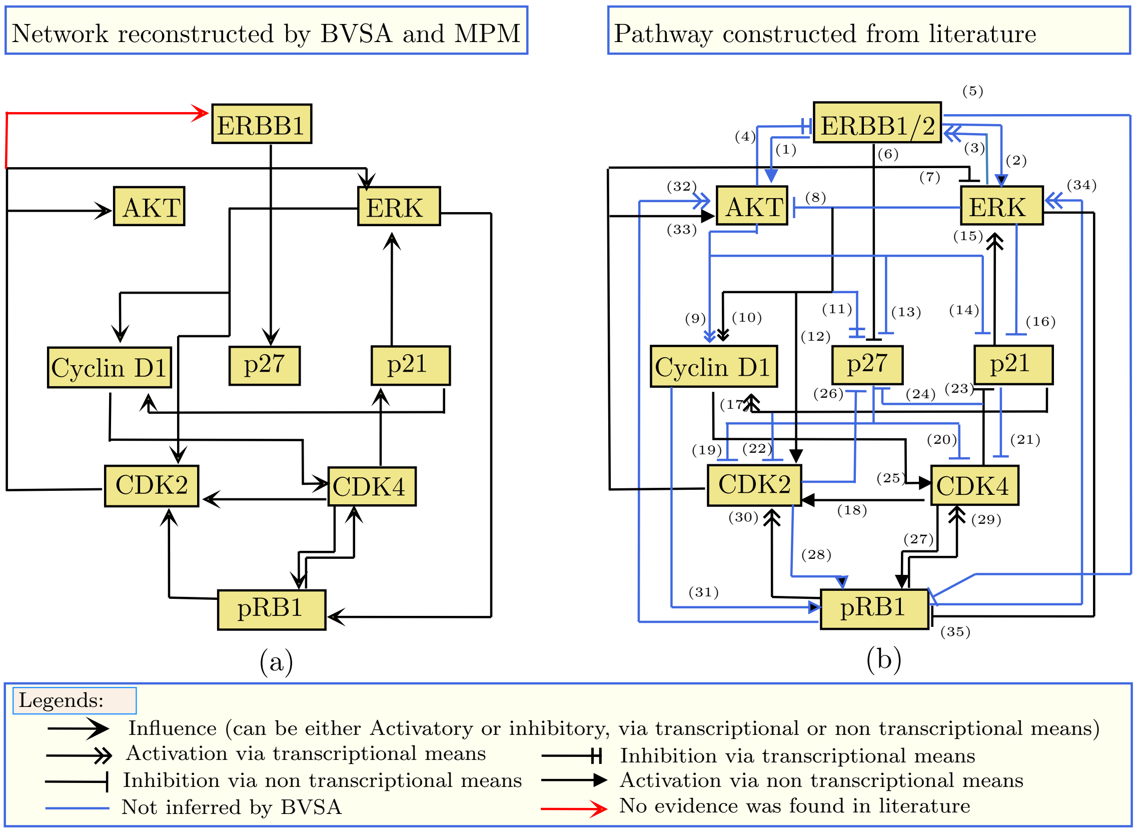

Figure S4: The ERBB-G1/S transition network inferred by the Median Probability Model
