# Supplementary material for: Integrating Bayesian variable selection with Modular Response Analysis to infer biochemical network topology
Source: BMC Syst Biol. 2013 Jul 6;7:57. doi: 10.1186/1752-0509-7-57 (PMC3726398; doi:10.1186/1752-0509-7-57)
Supplement: Additional file 11 — Figure S5. In this figure, we have shown the histograms of the connection coefficients of the ERBB regulated G1/S transition pathway as calculated by the stochastic MRA algorithm. [file 1752-0509-7-57-S11.pdf]

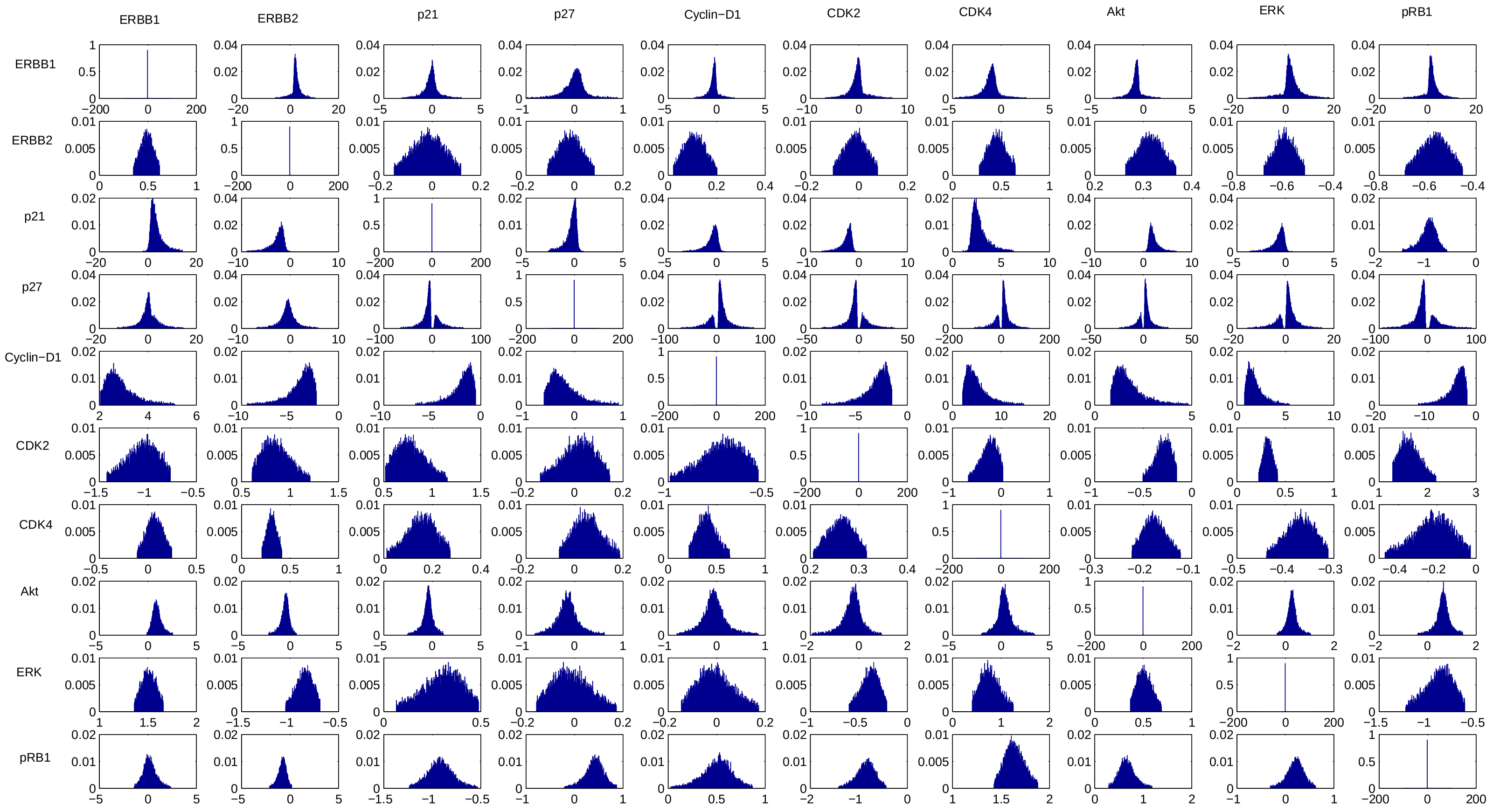

Figure S5: The histograms of the connection coefficients of the ERBB regulated G1/S transition pathway. The rows represent the regulated proteins and the columns represent the regulators. To explain, the second diagram from the left in the top row represents the histogram of the connection coefficient corresponding the interaction which indicates how ERBB1 is regulated by ERBB2. Similarly, the third from left in the same row represents the histogram of the connection coefficient corresponding to the interaction which indicates how ERBB1 is regulated by p21, and so on. The X-axis of each small diagram represents the values of the corresponding connection coefficients, and the Y-axis represents the frequency of occurrence.
